# Role of Epigenetic Factors in Determining the Biological Behavior and Prognosis of Hepatocellular Carcinoma

**DOI:** 10.3390/diagnostics14171925

**Published:** 2024-08-31

**Authors:** Sami Akbulut, Zeynep Kucukakcali, Tevfik Tolga Sahin, Cemil Colak, Sezai Yilmaz

**Affiliations:** 1 Liver Transplant Institute and Department of Surgery, Faculty of Medicine, Inonu University, 44280 Malatya, Turkey; tolga.sahin@inonu.edu.tr (T.T.S.); sezai.yilmaz@inonu.edu.tr (S.Y.); 2 Department of Biostatistics and Medical Informatics, Faculty of Medicine, Inonu University, 44280 Malatya, Turkey; zeynep.tunc@inonu.edu.tr (Z.K.); cemil.colak@inonu.edu.tr (C.C.)

**Keywords:** hepatocellular carcinoma, genetics, epigenetics, bioinformatic analysis, biological behavior, prognosis

## Abstract

Background: The current study’s objective is to evaluate the molecular genetic mechanisms influencing the biological behavior of hepatocellular carcinoma (HCC) by analyzing the transcriptomic and epigenetic signatures of the tumors. Methods: Transcriptomic data were downloaded from the NCBI GEO database. We investigated the expression differences between the GSE46444 (48 cirrhotic tissues versus 88 HCC tissues) and GSE63898 (168 cirrhotic tissues versus 228 HCC tissues) data sets using GEO2R. Differentially expressed genes were evaluated using GO and KEGG metabolic pathway analysis websites. Whole genome bisulfite sequencing (WGBS) and Methylated DNA Immunoprecipitation Sequencing (MeDIP-Seq) data sets (26 HCC tissues versus 26 adjacent non-tumoral tissues) were also downloaded from the NCBI SRA database. These data sets were analyzed using Bismark and QSEA, respectively. The methylation differences between the groups were assessed using functional enrichment analysis. Results: In the GSE46444 data set, 80 genes were upregulated, and 315 genes were downregulated in the tumor tissue (HCC tissue) compared to the non-tumor cirrhotic tissue. In the GSE63898 data set, 1261 genes were upregulated, and 458 genes were downregulated in the cirrhotic tissue compared to the tumor tissues. WGBS revealed that 20 protein-coding loci were hypermethylated. while the hypomethylated regions were non-protein-coding. The methylated residues of the tumor tissue, non-tumorous cirrhotic tissue, and healthy tissue were comparable. MeDIP-Seq, conducted on tumoral and non-tumoral tissues, identified hypermethylated or hypomethylated areas as protein-coding regions. The functional enrichment analysis indicated that these genes were related to pathways including peroxisome, focal adhesion, mTOR, RAP1, Phospholipase D, Ras, and PI3K/AKT signal transduction. Conclusions: The investigation of transcriptomic and epigenetic mechanisms identified several genes significant in the biological behavior of HCC. These genes present potential targets for the development of targeted therapy.

## 1. Introduction

Hepatocellular carcinoma (HCC) ranks as the sixth most common cancer, and it is the third most common cause of cancer-related mortality. Chronic liver disease is the major risk factor for the development of HCC [1]. Hepatocellular cancer has an inflammatory tumor microenvironment, and the chronic inflammatory state promotes tumor growth, progression, and immune evasion [2]. Annually, there are 906,000 newly diagnosed cases of primary liver cancer, and 830,000 patients lose their lives due to this disease. Primary liver cancer includes HCC and cholangiocarcinoma, but there are various rarer tumor types as well [3,4]. It is a heterogeneous tumor containing different phenotypes of tumor cells with different biologic behavior. The demographic and biological characteristics of HCC vary significantly as well. The incidence of HCC has a geographic and racial variance [5]. All these may be explained by the fact that there are differences in the distribution of various risk factors such as viral hepatitis, and environmental carcinogens (aflatoxins, etc.) [4,5,6,7]. In addition, many genetic changes during the carcinogenesis process of HCC result in a heterogeneous tumor with various prognoses [8].

Mutations in various genes alone cannot fully account for this heterogeneity. Additionally, cancer stem cells play an important role in the biological behavior of HCC such as progression and resistance to treatment [9,10]. Also, epigenetic changes that alter the gene function and the transcriptome contribute to hepatocarcinogenesis, progression, and metastasis [11,12,13]. Epigenetic modifications such as methylation, histone acetylation/methylation, and the non-coding RNAs change the conformation of the chromatin and result in changes in the function and expression of various genes without changing the nucleotide sequence of the genome. Epigenetic changes are heritable alterations of gene function. Epigenetic alterations are the link between environmental factors and the carcinogenesis process [14]. The liver is continuously exposed to environmental stressors such as dietary xenobiotics, viral and other infective agents, and the products of changing gut microbiota [15]. These environmental changes may lead to mutations in the hepatocytes. Together with the epigenetic changes, these environmental stressors and the resulting mutations lead to neoplastic transformation [16,17]. As the genetic and epigenetic changes accumulate, this leads to changes in the expression of tumor suppressor genes and protooncogenes, leading to HCC development, progression, and metastasis [18,19]. Epigenetic changes such as DNA methylation have an integral role in hepatocarcinogenesis [20]. Studies have shown that the metastatic and recurrent foci share similar methylation and mutation profiles as the primary HCC niche. Therefore, DNA methylation can be used as a marker to determine prognosis and predict the recurrence risk of patients with HCC [21,22]. Bioinformatics uses specific software for mass data analyses of the genomic data available on various open-source databases [4,14,23,24,25]. Bioinformatics is vital for integrating genetic and phenotypic characteristics of all cancers. This provides opportunities for developing individualized therapeutic options [4,14,23,24,25]. 

Clinical HCC research does not consider the molecular and genetic factors that shape the biological behavior of the tumor. There is a gap in our knowledge regarding genetic and epigenetic shifts in the progression of HCC. Moreover, there is a significant need for comprehensive studies comparing HCC and non-tumoral liver tissues to reveal distinct genetic and epigenetic characteristics of HCC. This will be important for the development of targeted therapies [26,27]. The present study aimed to evaluate the genetic and epigenetic changes in HCC using open-source databases. Furthermore, we tried to investigate the functional results of these changes from the perspective of cancer biology.

## 2. Materials and Methods

### 2.1. Microarray Data Analysis for Transcriptome Profiling

#### 2.1.1. Data Set and Basic Features

Various researchers have previously performed high-yield microarrays on HCC. We used these studies for advanced bioinformatic and biostatistical analyses to determine the potential genetic and metabolic pathways that play a role in the development and progression of HCC [28,29]. In the present study, we have analyzed two microarray-based transcriptomic sets. The basic characteristics of these data sets are summarized in Table 1.

These two data sets provide important molecular insights regarding HCC. They have advantages in both sample size and the experimental design. On the other hand, the two studies lack sufficient information about gene ontology, metabolic pathways, gene set enrichment, prominent gene promoter regions, transcription factors, protein-to-protein interactions, gene-to-gene interaction networks, and potential gene hubs. We aimed to fill this gap. In addition, we evaluated the methylation profile in HCC.

#### 2.1.2. Transcriptome Profiling with Microarrays

All microarray transcriptome data sets were downloaded in “.cel” and “.txt” format from the NCBI GEO (NCBI: National Center for Biotechnology Information, GEO: Gene expression omnibus) database (https://www.ncbi.nlm.nih.gov/gds (accessed on 1 October 2022)). All analyses were performed with the Bioconductor software package (version 3.19) from the R platform (https://bioconductor.org/ (accessed on 1 October 2022)) (Heidelberg, Germany). The “GSE46444” [28] and “GSE63898” [29] data sets were analyzed separately because they had different experimental designs. Also, these studies used different microarray platforms and probes. We used consensus gene names and IDs that were determined by the biomaRt v2.52.0 package (Heidelberg, Germany) [30]. As a result, regardless of the difference in the microarray platforms, all gene names were stated by the human reference genome (GRCh38). Background corrections for all microarray data sets, log2 conversions, and quantile normalizations were performed using the “Robust Multichip Average (RMA)” method within the affy v1.75.0 [31] package. The genes that were expressed differently between the “GSE46444” and “GSE63898” data sets were determined using the limma v3.52.4 [32] package (limma v3.52.4) (fold change, log2FC > 1, *p* < 0.05, Benjamini and Hochberg False Discovery Rate). These criteria are valid for both upregulated and downregulated genes. The principal component analyses of the gene expressions among the groups and determination of the gene clusters were performed and schematized using the ClustVis [33] internet tool (open source and available from http://biit.cs.ut.ee/clustvis/ (accessed on 1 October 2022)). The gene expression differences between the groups were schematized (such as volcano plot, MA plot, heatmap, etc.) using the R software package (mostly the ggplot2). The gene ontology (GO) enrichment and molecular pathway analyses such as biologic process (BP), molecular function (MF), and cellular component (CC) for genes that showed differential expression were performed using ShinyGO v0.61 (http://bioinformatics.sdstate.edu/go/ (accessed on 1 October 2022)) software and also, g: Profiler (http://biit.cs.ut.ee/gprofiler/ (accessed on 1 October 2022)), GSEA (v.3.0 or higher), Cytoscape (v.3.6.0 or higher) and EnrichmentMap (v.3.0 or higher) software as defined by Reimand and colleagues [34]. The protein-to-protein interactions were determined by using the STRING v11.5 [35] internet tool. Gene set enrichment analyses were performed using the GeneCodis4 [36] software package. Gene co-expressions were performed using the CoExp (https://rytenlab.com/coexp (accessed on 1 October 2022)) [37] and CEMiTool (https://cemitool.sysbio.tools/ (accessed on 1 October 2022)) [38] software packages. 

### 2.2. Bioinformatics Analyses of Whole Genome Bisulfite Sequencing (WGBS)

In the present study, we analyzed the data set of the studies that performed WBGS in tumor and non-tumor tissues of the patients with HCC. The data set in the “.fastq” format was downloaded from the NCBI Sequence Read Archive (https://www.ncbi.nlm.nih.gov/sra (accessed on 1 October 2022)) database. The database keywords for the search were [“hepatocellular carcinoma” + “liver tissue” + “cirrhosis”+ “adjacent noncancerous liver tissue” + “human”]. The studies that matched with [“cell” + “cell line” + “murine” + “mouse” + “rat”] keywords were excluded from the analyses. Initially, to obtain high-quality reads, the raw data were filtered to prepare for the study. The filter process was performed by using the Trimmomatic v0.39 [39] and fast v0.20.0 [40] software. The steps for filtration included (i) removal of the adaptor sequences; (ii) filtration of the low-quality readings (Phred’s score < Q20); (iii) removal of the base pair length readings less than 50 bp; and (iv) removal of the low quality of reading of the first 5 bp. The readings before and after the filer process were schematized using the FASTQC v0.11.9 (https://www.bioinformatics.babraham.ac.uk/projects/fastqc/ (accessed on 1 October 2022)) software and a quality control file was formed by gathering the MultiQC v1.6 [41] intermediary files. The data summarized in Table 2 were chosen and used for the WGBS analyses of our study. The complete data set is presented as an Excel file in Supplementary Materials. The concise version of the data was obtained using the SeqKit v2.3.1 software [42]. 

The high-quality data obtained after the filter process were matched with the reference human genome using the Bowtie2 v2.5.0 software [43]. The genome-matched data in the “.sam” format were converted to the “.bam” format using the Samtools v1.16.1 software [44] and were prepared for methylation analysis. The recall of the genome-wide methylated areas was performed by using the widely accepted Bismark v0.24.0 software [45]. 

The differences in the methylation profile among the groups were assessed using the DSS v2.46.0 Bioconductor (http://www.bioconductor.org/packages/release/bioc/html/DSS.html (accessed on 1 October 2022)) package. The locations with different methylation profiles (either hypomethylated or hypermethylated) were extracted using the Bedtools v2.30.0 [46] software and saved as a separate file. The annotation of these files was performed using the Biomart (https://www.ensembl.org/info/data/biomart/index.html (accessed on 1 October 2022)) web tool. The GO categories, including the BP, MF, and CC, were analyzed using the ShinyGO v0.76.3 [47] web tool. 

### 2.3. Bioinformatics Analyses of MeDIP-Sequencing

We performed additional bioinformatic analyses of the data set of the studies using the MEDIP-Seq methodology comparing the tumor and non-tumor tissue of the patients with HCC. We used the same search criteria with the WGBS analyses to produce a homogenous data set. The MEDIP-Seq data were downloaded from the NCBI Sequence Read Archive (https://www.ncbi.nlm.nih.gov/sra (accessed on 1 October 2022)) database in “.fastq” format. The data from 52 samples including 26 HCC tumor tissue and 26 adjacent non-tumor tissue were included in the analyses and are summarized in Table 3.

The data from the 52 MEDIP-Seq analyses were downloaded from the SRA database using the SRA Toolkit 3.0.3 (https://github.com/ncbi/sra-tools (accessed on 1 October 2022)) software to the workstations. The raw data were filtered using the Trimmomatic v0.39 [39] and fast v0.20.0 [40] software. The filtration process included (i) removal of the adaptor sequences, (ii) filtration of the low-quality readings (Phred’s score < Q20), (iii) removal of the base pair length readings less than 50 bp, and (iv) removal of the low-quality reading of the first 5 bp. The readings before and after the filtering process were schematized using the FASTQC v0.11.9 (https://www.bioinformatics.babraham.ac.uk/projects/fastqc/ (accessed on 1 October 2022)) software, and a quality control file was formed by gathering the MultiQC v1.6 [41] intermediary files. The high-quality data obtained after the filtering process were matched with the reference human genome using the Bowtie2 v2.5.0 software [43]. The genome-matched data in “.sam” format were converted to the “.bam” format using the Samtools v1.16.1 software [44] and were prepared for MEDIP-Seq analysis. The genome-wide methylated areas were recalled using the QSEA v1.24.0 Bioconductor package [48]. The differences among the groups were analyzed using the QSEA v1.24.0 Bioconductor package. The differences in the methylation profile of different gene loci (either hypomethylated or hypermethylated), methylation density of the CpG islands, and the functional annotations were performed using the Biomart (https://www.ensembl.org/info/data/biomart/index.html (accessed on 1 October 2022)) web tool. The GO components, including BP, MF, and CC, and the Kyoto Encyclopedia of Genes and Genomes (KEGG) metabolic pathway analyses were performed using the ShinyGO v0.76.3 [47] web tool. We also used the Annotatr v1.24.0 Bioconductor package for the annotations [49]. 

### 2.4. Ethical and Financial Aspects of the Study

The current study was approved by Inonu University Health Sciences Institute meeting number 44 organized on 5 October 2022 (Approval number: 2022/44-08-05). The institutional review board approved the study design on 23 May 2023 (Approval number: 3935). This study was supported and funded by the Inonu University Scientific Research Projects Coordination Unit (Project code: TYL-2022-3138). 

## 3. Results

### 3.1. Results of Transcriptome Profiling with Microarrays Analysis

**Figure 1:**
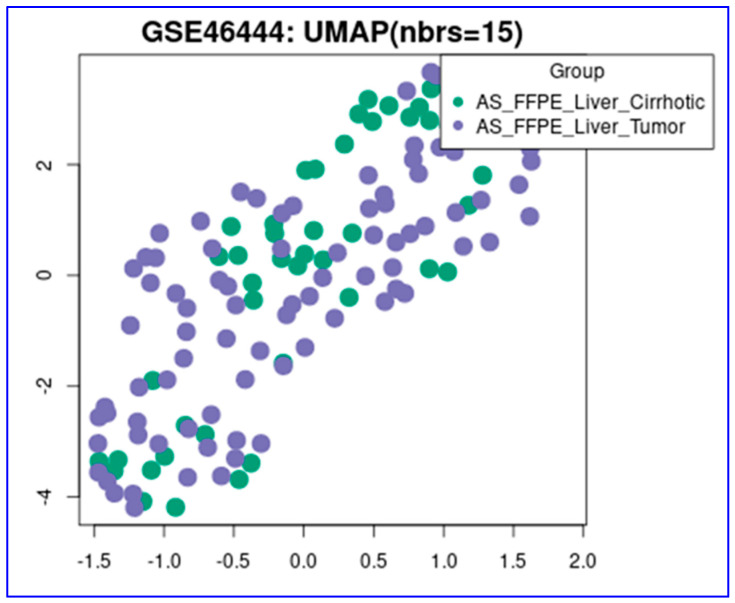
UMAP distribution of the transcriptomic profile of the cirrhotic and HCC tissues (green dots: cirrhotic tissues, purple dots: hepatocellular tissues) (GSE46444 data set) [HCC: hepatocellular carcinoma, UMAP: uniform manifold approximation and projection, AS-FFPE: archived sectioned formalin-fixed paraffin-embedded].

In the GSE46444 data set, 48 cirrhotic liver tissue and 88 HCC tumor tissue samples were compared for gene expression using transcriptomic microarray analysis on the FFPE tissues. Following normalization, sample heterogeneity was high regardless of the groups, as shown by the UMAP (Uniform Manifold Approximation and Projection) cluster analysis (Figure 1).

**Figure 2:**
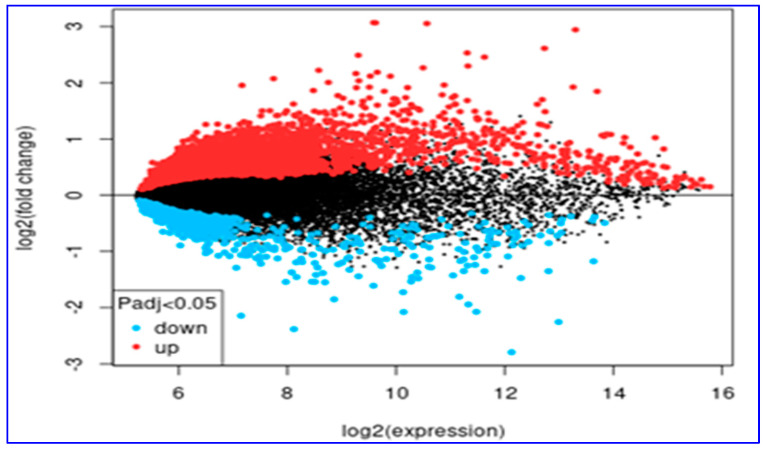
The MA graph of the upregulated (red dots) and downregulated (blue dots) genes in the cirrhotic tissues compared to HCC tissues. The black dots show the genes that did not differ in expression between the cirrhotic and HCC tissues (HCC: hepatocellular carcinoma).

Furthermore, gene expression levels changed significantly among the groups (↑ upregulated, downregulated) (|log2FC| > 1.0; *p* < 0.05). Four hundred and twenty-three genes were upregulated in cirrhotic livers compared to the HCC samples. On the other hand, in cirrhotic liver, 98 genes showed downregulation when compared to the HCC samples (Figure 2). The 25 genes that showed differential expression among the groups and those that encode a specific protein are summarized in Supplementary Tables S1 and S2. The heat map graphic of these genes is given in Supplementary Figure S1.

The functional annotations of the protein-coding genes using BP, MF, CC, and KEGG metabolic pathway enrichment analyses are summarized in Supplementary Tables S3–S6 and Supplementary Figures S2 and S3.

In the GSE63898 data set [29], 168 cirrhotic liver tissues were compared with 228 HCC tissues for gene expression levels using microarray transcriptomic analysis. Unlike the GSE46444 data set [28], UMAP cluster analysis following the normalization of the GSE63898 data set showed more successful and consistent discrimination (Supplementary Figure S4). Therefore, the data obtained from this data set seem more reliable and devoid of technical error. Similarly, we found significant changes in various gene expressions between the cirrhotic and HCC tissues in the GSE63898 data set (|log2FC| > 1.0; *p* < 0.05). When the cirrhotic and HCC tissues were compared, the expression of 1261 genes increased in the cirrhotic tissues, and the expression of 458 genes increased in the HCC tissues (Supplementary Figure S5). Twenty-five genes showed a difference in expression and took part in protein-coding. These protein-coding genes are summarized in Supplementary Tables S7 and S8. The heat map graphs are presented in Supplementary Figure S6.

The functional annotation and KEGG metabolic pathway analyses of 25 protein-coding genes that showed a difference in expression among the groups are summarized in Supplementary Tables S9–S12 and Supplementary Figures S7 and S8. 

### 3.2. Results of Whole Genome Bisulfite Sequencing Analysis

We analyzed the methylation profile of the whole genome. The results of the bisulfide analysis of the HCC tissue in comparison to normal and cirrhotic livers are summarized in Supplementary Tables S13 and S14.

Twenty hypermethylated gene loci were found in the HCC tissues. The results are summarized in Supplementary Table S13. On the other hand, the hypomethylated gene loci in HCC are summarized in Supplementary Table S14. The results of the GO analysis for the gene loci that showed differences in methylation profile are summarized in Supplementary Tables S15–S17 and Supplementary Figure S9. The results of the KEGG metabolic pathway analyses are given in Supplementary Table S18 and Supplementary Figure S10.

We also compared the changes in the methylation profile between the HCC and cirrhotic tissues using genome-wide bisulfite analyses. The top 20 genomic loci that showed differences in methylation profile are summarized in Supplementary Tables S19 and S20.

The results of the GO analyses regarding the gene loci that showed significant changes in the methylation profile in HCC tissues are summarized in Supplementary Tables S21–S23 and Supplementary Figure S11. The results of the KEGG metabolic pathway analyses showed no significant changes between the groups.

### 3.3. Results of MeDIP-Seq Analysis

The MeDIP-Seq analyses showed that 18,933 gene loci had significant changes in the methylation profile. The detailed evaluation of these gene loci showed that 15,380 were protein-coding regions, 3006 were long non-coding RNA (lncRNA), and the remaining 547 gene loci were pseudogenes. The top 25 gene loci that showed significant changes in the methylation profile in coding regions are summarized in Supplementary Tables S24 and S25. The MeDIp-Seq analyses of the 52 samples summarizing the CpG methylation density and enrichment are summarized in Supplementary Figure S12. There are significant differences in the methylation profile of the various gene loci between the groups (log2FC > 1; adj *p* value < 0.005 for hypermethylation; log2FC < −1; adj *p* value < 0.005 for hypomethylation). The DO and KEGG metabolic pathway analyses are summarized in Supplementary Figures S13 and S14. These results show that the methylation profile changed mainly in the RAP1, endocytosis, papillomavirus, and PI3AKT/AKT pathways. 

### 3.4. Summary of the Molecular and Metabolic Pathways of the Genes That Showed Differences in Expression and Methylation Profile

We summarized the common metabolic and molecular pathways that changed in all databases. The summary of our results is presented in Table 4. We formed this table by finding the common genes that changed in HCC in all databases analyzed in our study. Our results show that downregulated genes in HCC belong to lipid metabolism. In addition, vesicular transport is affected at the cellular level. On the other hand, upregulated genes in HCC belong to the components of steroid metabolism, oxidative stress, and inflammatory pathways. Major transcription factors such as the PI3AKT/AKT pathway were upregulated. This pathway is a major driver for the progression of the cell cycle and is responsible for the uncontrolled proliferation of cancer cells. The genes that showed changes in the methylation profile were mainly voltage-gated ion channels, extracellular matrix components, RNA, and DNA polymerases. We emphasize that these are common genes that changed expression or epigenetic modification profiles in different databases. 

## 4. Discussion

HCC is the most prevalent primary liver cancer globally. Moreover, it stands as the third leading cause of death related to cancer. Consequently, identifying potential genetic targets crucial to the pathogenesis of HCC is paramount. This is important for developing diagnostic and prognostic biomarkers as well as new therapeutic options [50,51]. The “-omics” analytical methodologies that involve genomic, transcriptomic, epigenomics, proteomics, and metabolomic analyses are currently cutting-edge research tools that play an important role in determining the molecular mechanisms of HCC and various other diseases that have a genetic basis [52]. Genome-wide association studies (GWAS) are effective tools for genomic, epigenetic, and gene function analyses in various diseases. In our study, we analyzed two data sets downloaded from the NCBI GEO database. As a result, we found significant changes in various gene expressions in HCC tissues compared to non-tumoral tissues. Also, we found epigenetic changes (such as changes in methylation profile) in promoter regions that play an important role in gene expression. We analyzed the methylation profile of the data sets using WGBS and MeDIP-Seq methodology. 

The microarray transcriptomic analysis of the GEO [GSE46444] data set [28] involving FFPE 83 HCC, and 47 cirrhotic liver tissues showed that 80 genes were downregulated and 315 were upregulated in HCC tissues. The most important genes that were downregulated in HCC tissues were ADH4, CNGB1, MGC10997, PROM1, MALAT1, GYG2, ARHGAP8, MT1F, VIPR1, and HEATR2. On the other hand, the top 10 genes that were upregulated in HCC tissues were ALG1L, PITX1, SPINK1, ACSL4, SLC26A6, CDKN2A, ITPKA, APOA2, ECT2, and NEK2. The ALG1L gene is related to the glycosyl transferase enzyme, and it has been reported that this gene and the product glycosyl transferase are related to the progression and early diagnosis of HCC tumors [53]. PITX1 is a transcription factor that controls the developmental process of mammals and is closely related to the embryonic development of many organs, including the extremities and the heart [54]. The PITX1 protein can be detected in serum samples, and it has been shown that it is a specific marker for the diagnosis of HCC in the early stages [55]. In addition, the SPINK1 protein is overexpressed in HCC which develops in patients with hemochromatosis, and it is shown that SPINK1 gene expression increases during the cirrhosis to HCC transition [56,57]. SPINK1 is overexpressed in metastatic HCC. However, since it is expressed in many tumors, it cannot be used as a diagnostic or a prognostic marker [58]. We encountered various problems during the analysis process. Especially in the GSE46444 data set [28], the cluster analysis showed that there is no significant difference. We believe this may have resulted from increased variation in the data obtained from the FFPE tissues in this data set. The RNA obtained from these tissues may have been fragmented, thia may have caused the variation. 

We have found that the ACSL4 gene is upregulated in HCC tissues. This has also been confirmed by other research indicating that it has an important physiologic role in sorafenib-dependent ferroptosis and as a biomarker for response to sorafenib therapy [59]. Furthermore, the ACSL4 gene has an active role in lipid metabolism, which is the regulation of de novo lipogenesis [60]. It performs these functions with SREBP1 and c-Myc. Especially ACSL4 has a strong correlation with SREBP1; for these reasons, these two markers may be strong diagnostic markers [61]. 

Another important point that should be emphasized regarding our findings is the upregulated expression of CDKN2A in HCC tissues. CDKN2A has important correlations with tumor-associated macrophages and immune infiltrates [61]. We found upregulation of Inositol-Trisphosphate 3-Kinase C (ITPKC), a gene that plays a role in various cellular processes. Our results showed that ECT2 was upregulated in HCC tissues. Studies have shown that ITPKC and ECT2 are upregulated in the tumorigenesis process and in the advanced stages of the tumors [56]. ITPKC activates the Ca2+/NFAT pathway and inactivates T-lymphocytes [62]. ITPKC overexpression is associated with an increased risk of liver metastasis in colorectal cancer [63]. Furthermore, ITPKC over-expression in triple-negative breast cancer was associated with reduced disease-free survival and poor prognosis [64]. To our knowledge, ours is one of the first studies to emphasize the importance of ITPKC in hepatocellular cancer. The ECT2/Rho pathway promotes the growth and metastasis of hepatocellular cancer and is upregulated in the advanced form of the disease [65]. 

The results of the microarray analysis on the GEO [GSE63898] data set [29] suggested that 1261 genes were downregulated and 458 were upregulated in HCC tissues. Among the genes that were downregulated in the HCC tissues were CXCL14, CLEC4G, ADAMTS13, FCN2, CRHBP, ANGPTL6, COLEC10, FCN3, PTH1R, and RSPO3. The most important genes that were upregulated in the HCC tissues were CAP2, TOP2A, ASPM, CCT3, KLHL12, SNX27, RACGAP1, PLVAP, FAM189B, and GOLPH3L. The CAP2 gene needs emphasis because, although its exact function in the organism is unknown, it interacts with adenylyl cyclase-associated protein and actin [66]. For this reason, it may have a role in tumor invasion and metastasis. The CAP2 gene was activated in both transcriptional and the translational stages of g. Studies have shown that nearly 80% of the HCC tissues show prominent CAP2 protein in immunohistochemical analysis [67]. 

The TP2A gene is located on Chromosome 17, and many studies have analyzed the role of this gene in HCC. TOP2A gene expression has been evaluated in HCC tissues and was upregulated in tumoral tissue [68]. A recent study has shown that TOP2A was a direct target of miR-144-3p and the expression of TOP2A was upregulated in patients with poor prognosis of the patients. The authors showed that miR-144-3p was the reason for the overexpression of TOP2A and resulted in the proliferation and metastasis of HCC cells [69]. Various studies have shown that the ASPM gene is upregulated in HCC tissues, and it is correlated with more aggressive tumors and reduced patient survival [70,71]. In vitro transfection studies on HCC cell lines showed that the knockdown of ASPM reduced the invasion, proliferation capabilities, and epithelial-to-mesenchymal transition of HCC cells [72]. The RACGAP1 gene was prominently upregulated in HCC. This gene has been extensively studied in different cancers, but in vivo and in vitro studies have only recently shown that RACGAP1 is over-expressed in HCC. In addition, it is in the downstream of RACGAP1 that it exerts its biological effects through the activation of the PI3K/AKT/CDK2 and PI3K/AKT/GSK3β/Cyclin D1 pathways [73]. In a recent study, it was shown that FAM189B is significantly upregulated in HCC tumoral tissues. The FAM189A1 and FAM189A2 genes from the same family showed no significant difference in expression between the tumoral and non-tumoral tissues [74]. Our study is unique because it emphasizes the importance of upregulated FAM189B expression in HCC tissues. In the present study, the whole genome bisulfite analyses showed that HCC tissues showed hypermethylation in 20 protein-coding loci. The hypomethylated regions were non-coding regions. Hypermethylation of the CPG moieties in the genome results in tissue-specific transcripts [75]. In HCC, various genes responsible for morphogenesis (embryonic organogenesis and musculoskeletal) and development (embryonic tissue, organogenesis, and neurogenesis) showed changes in methylation profile. The molecular functions of the genes with different methylation profiles were related to voltage-gated potassium channels, potassium channels, voltage-gated ion channels, transmembrane transporters of potassium, structural components of the extracellular matrix, and other gated channels. Recent studies have shown that SCN3B has important roles in tumor growth and metastasis in HCC tissues, and in vitro studies have shown that they regulate the expression of voltage-gated sodium channels, suppress the activity of the p53 gene and reduce apoptosis and increase proliferation in HepG2 cell lines [76]. Eag1 is extensively expressed in cirrhotic tissue in the preneoplastic stage and in HCC tumor tissues. Therefore, it can be a diagnostic biomarker for HCC [77]. An important biological function of these genes is DNA-binding activity. DNA-binding protein A (DBPA) has a role in inflammation-related hepatocarcinogenesis. Studies have shown that it is correlated with advanced-stage HCC [78,79,80,81,82,83]. The changes in the methylation profile of the genes are important for understanding hepatocarcinogenesis at the molecular level, regardless of the different types of HCC.

The molecular function enrichment studies were performed on the protein-coding loci that showed changes in the methylation profile. These genes were mTOR, focal adhesion and related pathways, RAP1, phospholipase D, Ras, and PI3K/AKT signal transduction pathways. The changes in the methylation profile of the genes located on the PI3K/AKT pathway affect cell growth, proliferation, cellular metabolism, cell motility, cell survival, and apoptosis [84]. Studies have shown that Neat1 is the target of mTOR, which plays an important role in cellular glucose metabolism in HCC. The changes in the glucose metabolism due to the Neat1/mTOR pathway are defined as the” Warburg effect”. Also, studies have shown that mTOR could be a good target for developing new therapies for HCC [85]. A study has shown that CD73, an enzyme that converts AMP to adenosine, activates the PI3K/AKT pathway and is a prognostic marker for HCC [86]. The present study aims to evaluate the role of these genes in different biological processes and methylation analyses; we used both WGBS methylation analysis and MEDIP-Seq analysis [87]. We used the advantages of these two techniques to increase the reliability of our results. WGBS determines methylation changes in single nucleotide positions; on the other hand, MEDIP-Seq uses antibody-mediated determination of methylated areas (especially on CpG islands) on the genome, and it can determine changes in the methylation profiles of multiple nucleotide positions with great accuracy [88]. 

We downloaded the genomic data of HCC and non-tumor tissues from the NCBI SRA open-source database. The MEDIP-Seq analysis determined the methylation profile of the CpG-rich regions, which resulted in the determination of protein-coding regions of the genome. Changes in the methylation profile of the genes change the expression levels and result in changes in the biological behavior of HCC. In the context of HCC, this study addresses critical gaps in our understanding of the molecular genetics of HCC. Epidemiologic studies have shown that incidence and mortality are significantly affected by geographic and demographic changes. This observation emphasizes the importance of our research. The investigation of the genetic and epigenetic profile of HCC is particularly valid given the disease’s complexity and heterogeneity. The variations in biological behavior and prognosis of HCC in different populations suggest a strong influence of genetic and epigenetic factors. Our research contributes to this understanding by using open-source databases to examine these molecular changes and their functional implications in cancer biology. This research strategy is ideal to identify molecular mechanisms related to HCC which are important for new treatment modalities. The significant correlation of HCC with cirrhosis further underscores the importance of understanding these molecular changes, especially in high-risk populations. Therefore, our results are a crucial step towards unraveling the complex genetic and epigenetic landscape of HCC, which could lead to groundbreaking advancements in targeted therapies and improved patient outcomes.

We found that genes related to vesicular transport were downregulated in HCC. A recent study emphasized the importance of vesicular transport-related genes in immune infiltration in the HCC microenvironment and response to treatment with immune checkpoint inhibitors [89]. Our results support this observation because this important gene family was downregulated in patients with cancer, which accelerated the hepatocarcinogenesis process. On the other hand, generally, molecular research on lipid metabolism suggests that cancer cells upregulate the components of lipid metabolism to grow and proliferate [90,91]. Our results contradict these observations. We believe the stage of the tumor determines the dominant energy metabolism. The databases we used in our study have a heterogeneous group of patients in terms of stage of HCC, which may have caused this discrepancy. 

The present study has some limitations. We used the transcriptomic data obtained from other studies. From this perspective, our results are based on retrospective analyses. Furthermore, we did not perform any experimental procedure, and the results of this study lack fundamental experimentally and clinically validated data, which is an important limitation of this study. However, this is the nature of bioinformatic studies. The retrospective nature of the studies performed separately can raise quality concerns. We have tried to overcome these by selecting high-quality data from the databases. The details of the acquisition of our data are explained in detail in the Materials and Methods section. Another limitation of the present study is that we have not correlated the results of the genetic and epigenetic changes with the prognosis and survival data of the patients because the two databases we used did not include data regarding the survival and prognosis of the patients. On the other hand, our results provide important information regarding epigenetic changes in hepatocellular cancer. A single study cannot fill the gap regarding a detailed subject such as genetic and epigenetic changes in HCC. However, our results are genuine, and our results regarding vesicular transport genes are one of the first studies emphasizing this point [89]. It is our opinion that our data will guide future studies that can investigate the prognostic significance of the genetic and epigenetic changes that we have emphasized in HCC.

## 5. Conclusions

In conclusion, our results have shown that transcriptomic and methylation-related epigenetic mechanisms in HCC contribute to our understanding of tumor biology. Understanding tumor biology enables us to develop markers for diagnosis and prognostication. Moreover, it leads to the determination of potential targets for the development of new therapeutic approaches.

## Figures and Tables

**Table 1:** Basic information regarding the transcriptomic analyses of HCC: (a) the Illumina Human Whole Genome DASL HT; and (b) the Affymetrix Human Genome U219 Array (HG-U219) platforms were used.

GEO Access ID	Platform ID	Number of Samples	The Aim of the Study
GSE46444 [28]	GPL13369 (a)	136	Transcriptomic analyses were performed using the archived FFPE and fresh HCC and non-tumorous cirrhotic tissues that were compared with each other. A total of 88 FFPE HCC versus 48 fresh cirrhotic liver tissue samples were analyzed. The results showed that old FFPE HCC tissue could be used in transcriptomic analysis for the diagnosis and classification of the disease. The study provided no data regarding the molecular processes of HCC.
GSE63898 [29]	GPL13667 (b)	396	The sample number used in this study is high, with 228 HCC and 168 cirrhotic liver tissue samples. Transcriptomic microarray analyses were performed. The important characteristic of the study was the presence of microarray transcriptomic analyses together with methylation analysis. The study provides detailed data, including epi-driver genes, and are correlated with the survival data of the patients.

FFPE: formalin-fixed paraffin-embedded; HCC: hepatocellular carcinoma.

**Table 2:** The transcriptomic data of the samples obtained from the NCBI SRA database for WGBS analysis are characterized.

Tissue Type	Number of Samples (*n*)	Average Number of Measurements	Mean Number of Nucleotides
Tumor (HCC) tissue	34	10,690,595	744,661,764
Healthy liver tissue	27	5,132,889	518,421,848
Adjacent non-tumor liver tissue	7	23,028,257	1,278,260,601
Cirrhotic liver tissue	8	30,104,184	1,535,313,410

HCC: hepatocellular carcinoma.

**Table 3:** The results of the MEDIP-Seq analyses of the tumor and the non-tumor liver tissues.

Tissue Type	Number of Samples (*n*)	Average Number of Measurements	Mean Number of Nucleotides
Tumor (HCC) tissue	26	22,254,325	836,263,008
Adjacent non-tumor liver tissue	26	23,025,232	865,227,639

HCC: hepatocellular carcinoma.

**Table 4:** Summary of pathways and molecular functions of the genes that changed expression and methylation profile in HCC. HCC: hepatocellular cancer, PI3K: Phosphoinositide 3-kinase, Akt: Protein kinase B, DNA: Deoxyribonucleic acid, RNA: Ribonucleic acid.

Downregulated Pathways in HCC	Upregulated Pathways in HCC	Molecular Function Genes That Had a Change in Methylation Profile in HCC
Olefinic compound metabolic processes	Arachidonic acid epoxygenase activity	Voltage-gated potassium channel activity
Monocarboxylic acid metabolic processes	Aromatase activity	Potassium channel activity
Carboxylic acid metabolic processes	Steroid hydroxylase activity	Voltage-gated ion channel activity
Oxoacid metabolic processes	Oxidoreductase activity	Potassium ion transmembrane transporter activity
Lipid metabolic processes	Monooxygenase activity	Extracellular matrix structural constituent
Blood microparticle	Heme binding	Gated channel activity
Secretory granule lumen	Tetrapyrrole binding	Ion channel activity
Cytoplasmic vesicle lumen	Iron ion binding	Cis-regulatory region sequence-specific DNA binding
Vesicle lumen	Transition metal ion binding	RNA polymerase II cis-regulatory region sequence-specific DNA binding
	Drug metabolism	Channel activity
	Mineral absorption	Transcription cis-regulatory region binding
	Retinol metabolism	Transcription regulatory region nucleic acid binding
	Metabolism of xenobiotics by cytochrome P450	DNA-binding transcription factor activity. RNA polymerase II-specific
	Chemical carcinogenesis	Sequence-specific DNA binding
	Fatty acid degradation	Sequence-specific double-stranded DNA binding
	Bile secretion	RNA polymerase II transcription regulatory region sequence-specific DNA binding
	Complement and coagulation cascades	DNA-binding transcription factor activity
	PI3K-Akt signaling pathway	Calcium ion binding
	Pathways in cancer	Double-stranded DNA binding

## Data Availability

The data sets analyzed during the current study are available from the corresponding author on reasonable request.

## Supplementary Materials

The following supporting information can be downloaded at: https://www.mdpi.com/article/10.3390/diagnostics14171925/s1.
