# Supplementary material for: Role of Epigenetic Factors in Determining the Biological Behavior and Prognosis of Hepatocellular Carcinoma
Source: Diagnostics (Basel). 2024 Aug 31;14(17):1925. doi: 10.3390/diagnostics14171925 (PMC11394249; doi:10.3390/diagnostics14171925)
Supplement: Supplementary file 1 [file diagnostics-14-01925-s001.zip › Suppl Figures revised.pdf]

diagnostics

MDPI

Role of Epigenetic Factors in Determining the Biological Behavior and Prognosis of Hepatocellular Carcinoma

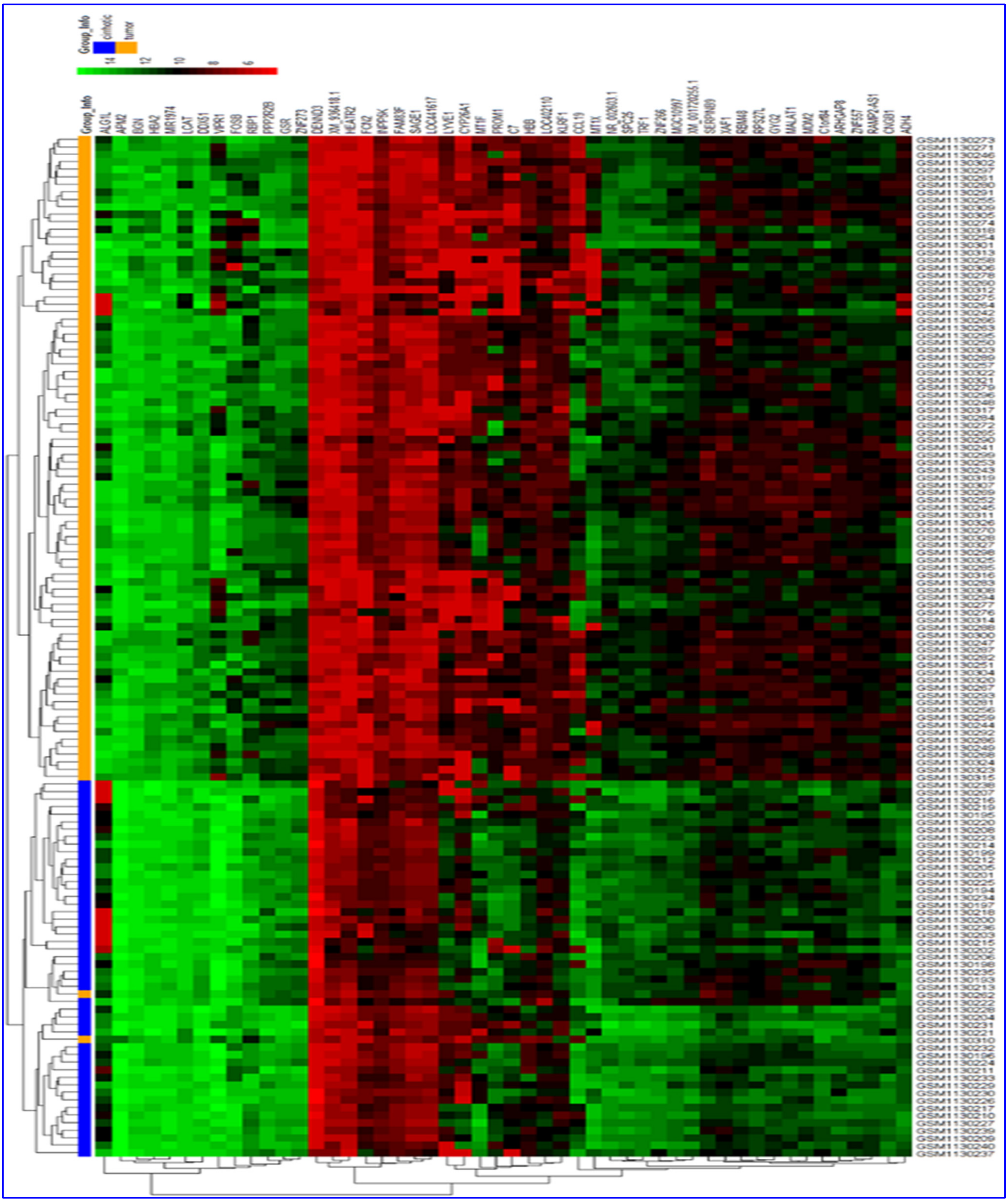

Suppl Figure 1: The heat-map graph of the first 50 genes that differed in expression between the samples from the cirrhotic and HCC tissues (GSE46444 data set) (HCC: Hepatocellular carcinoma).

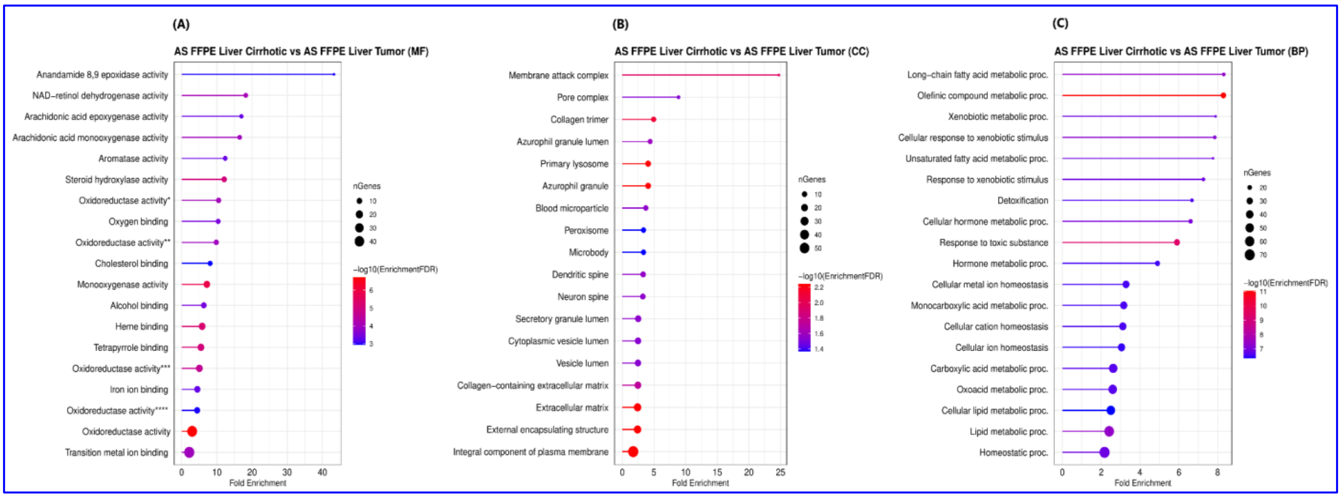

Suppl Figure 2: The BP, MF, and CC enrichment graph of the genes that changed in expression among the samples from the cirrhotic and HCC tissues (GSE46444 data set) [GO: gene ontology, BP: biologic process, MF: molecular function, and CC: cellular component, AS-FFPE: Archived sectioned formalin-fixed paraffin-embedded, HCC: Hepatocellular carcinoma].

A

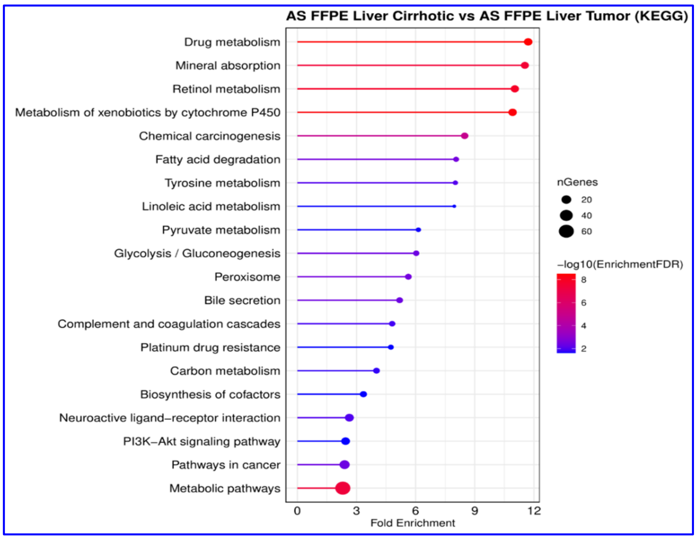

Suppl Figure 3: The metabolic pathway enrichment graph of the genes that changed in expression among the cirrhotic and HCC tissues (GSE46444 data set) [KEGG: Kyoto Encyclopedia of Genes and Genomes, AS-FFPE: Archived sectioned formalin-fixed paraffin-embedded, HCC: Hepatocellular carcinoma].

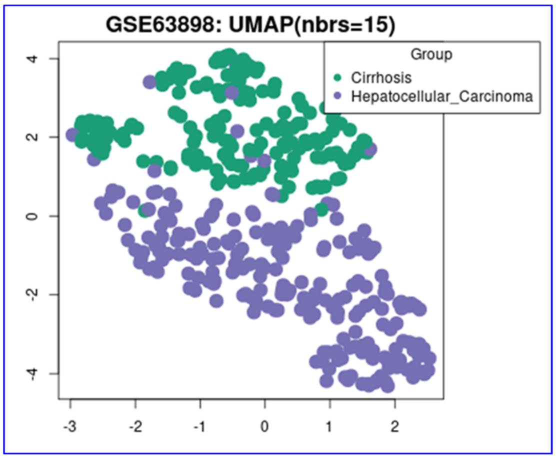

Suppl Figure 4: The UMAP distribution of the transcriptomic profile of the cirrhotic and HCC tissues (Green dots: cirrhotic samples, purple dots: hepatocellular cancer samples) (GSE63898 data set) [UMAP: Uniform manifold approximation and projection, HCC: Hepatocellular carcinoma].

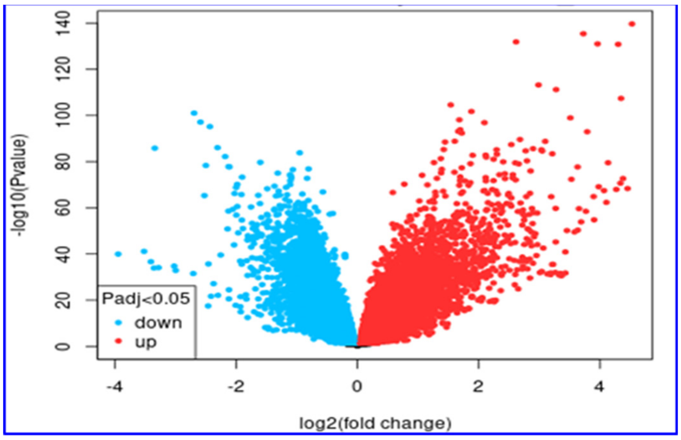

Suppl Figure 5: The Volcano graph of the genes that were upregulated (red dots) and downregulated (blue dots) among the samples from the cirrhotic and HCC tissues [HCC: Hepatocellular carcinoma].

#

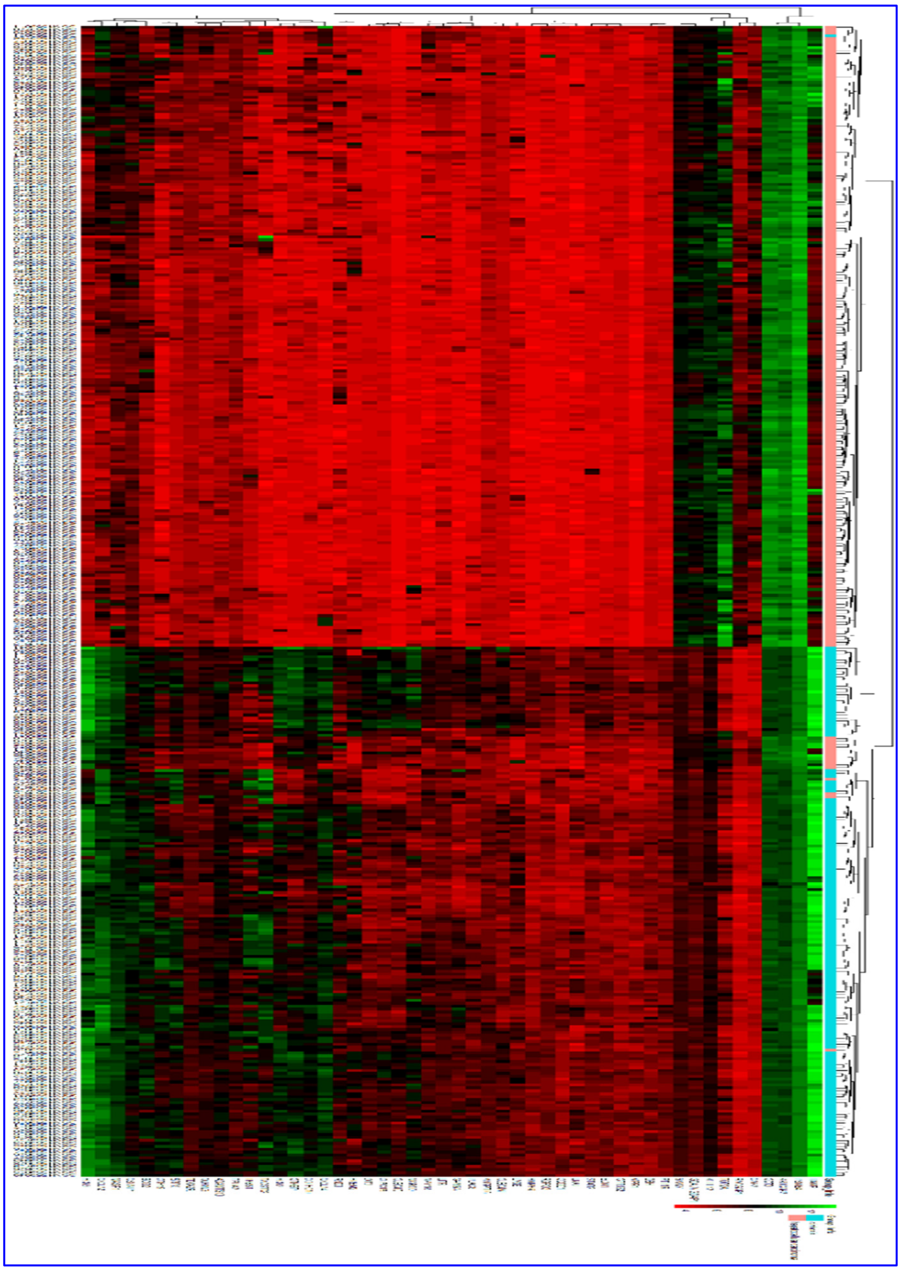

Suppl Figure 6: The heat-map graph summarizing the first 100 genes that differed in expression among the samples from cirrhotic and HCC tissues (GSE63898 data set) [HCC: Hepatocellular carcinoma].

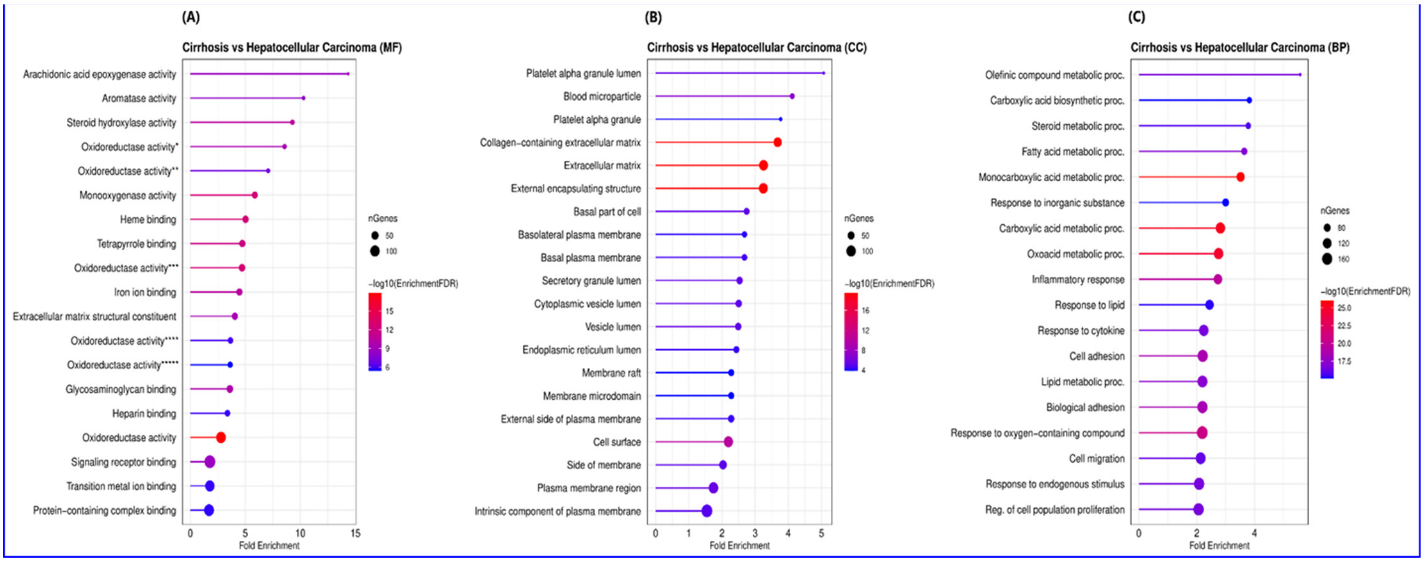

Suppl Figure 7: The BP, MF and CC enrichment graphs summarizing the differences between the samples from the cirrhotic and HCC tissues (the GSE63898 data set) [BP: biologic process, MF: molecular function, and CC: cellular component, HCC: Hepatocellular carcinoma].

#

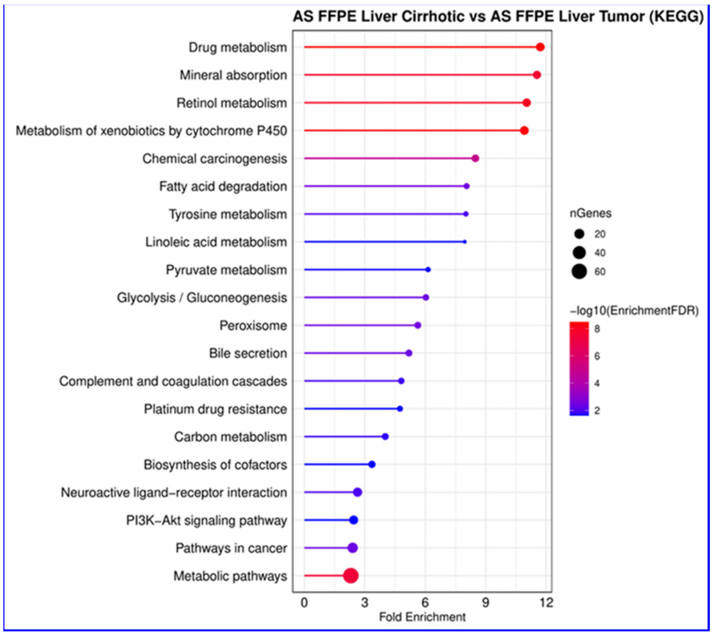

Suppl Figure 8: Summary of the differences in metabolic enrichment analyses between the samples from the cirrhotic and HCC tissues (the GSE63898 data set). [KEGG: Kyoto Encyclopedia of Genes and Genomes, AS-FFPE: Archived sectioned formalin-fixed paraffin-embedded, HCC: Hepatocellular carcinoma].#

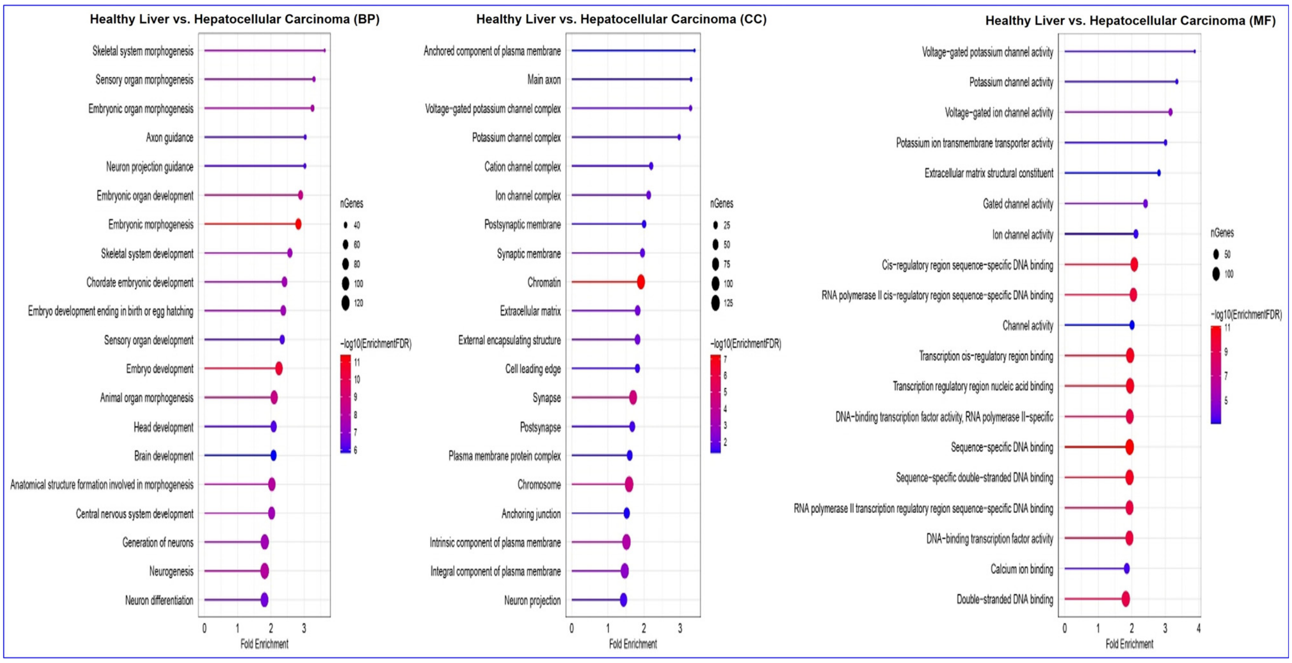

Suppl

Figure 9: The summary of the GO functional annotation of the gene loci that showed different methylation profiles in HCC tissues compared to healthy liver tissues [GO: gene ontology, BP: biologic process, MF: molecular function, and CC: cellular component, HCC: Hepatocellular carcinoma].

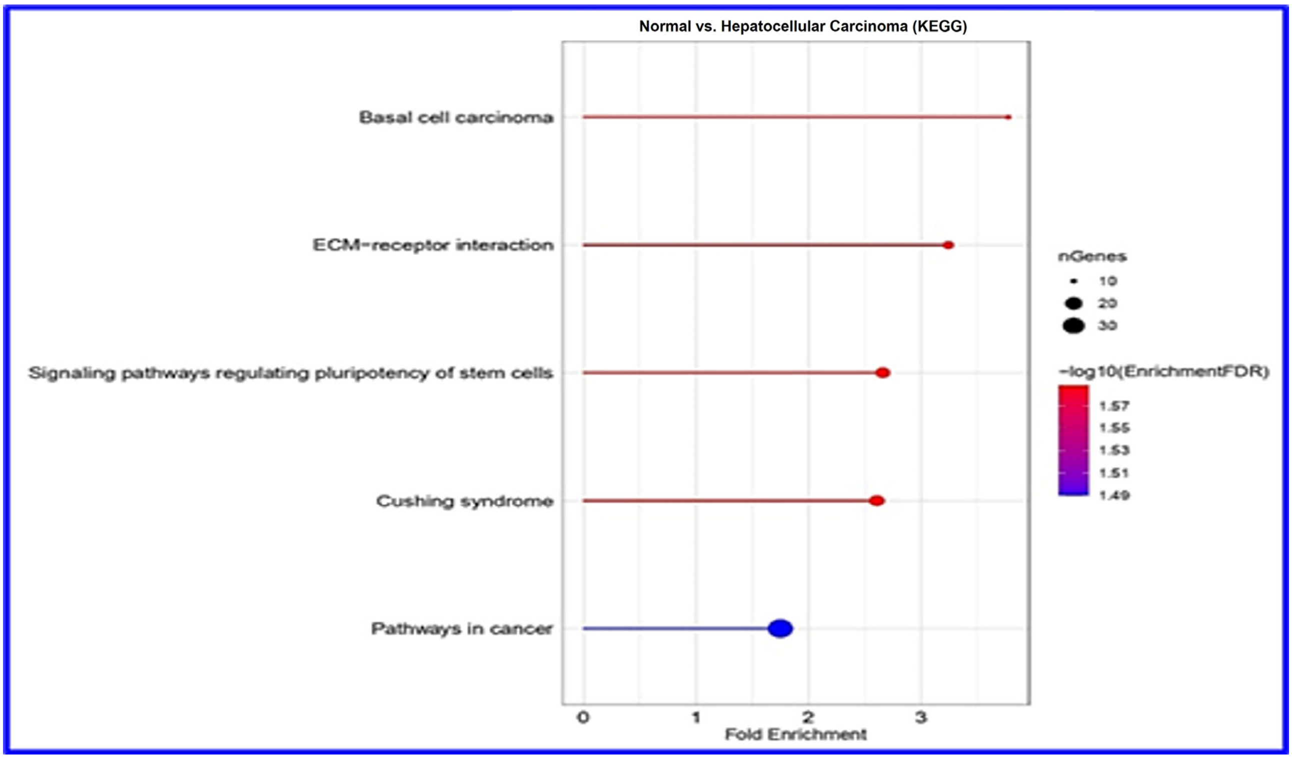

Suppl Figure 10: The summary of the KEGG metabolic pathway annotation of the gene loci that showed different methylation profiles in HCC tissues compared to healthy liver tissues. [KEGG: Kyoto Encyclopedia of Genes and Genomes, HCC: Hepatocellular carcinoma].#

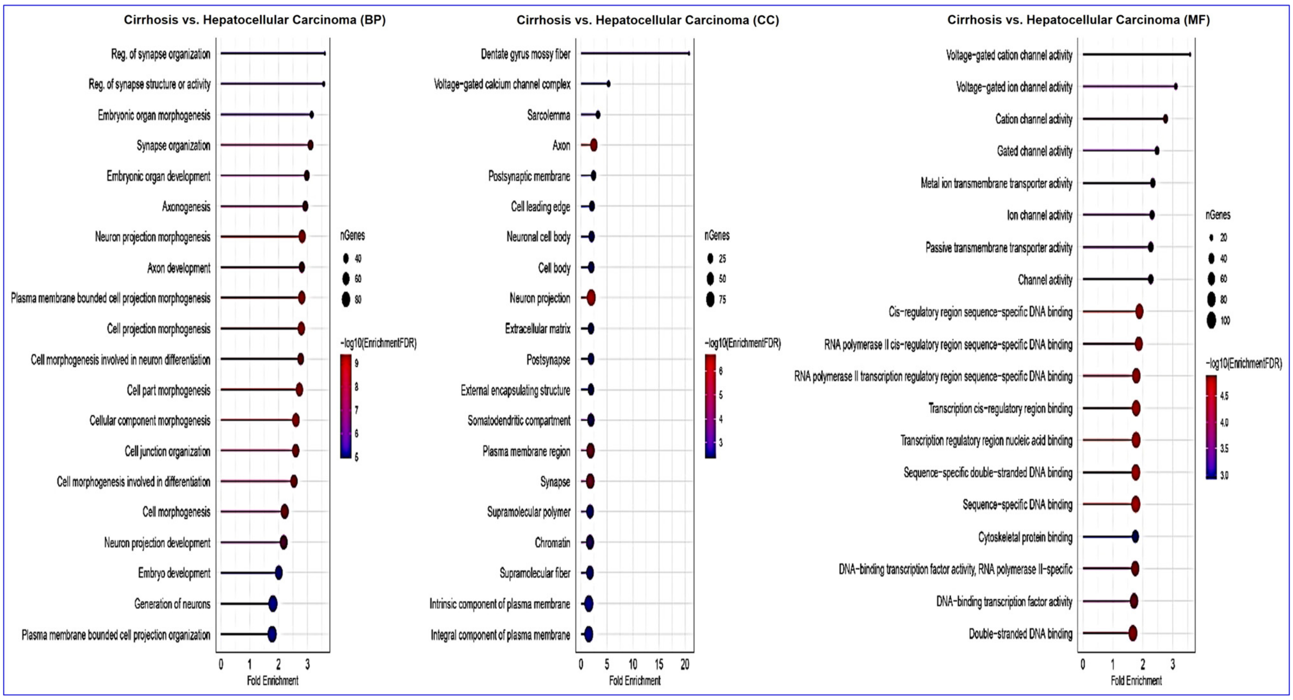

Suppl Figure 11: The summary of the GO functional annotation of the gene loci that showed different methylation profiles in HCC tissues compared to cirrhotic tissues. [GO: gene ontology, BP: biologic process, MF: molecular function, CC: cellular component, HCC: Hepatocellular carcinoma].

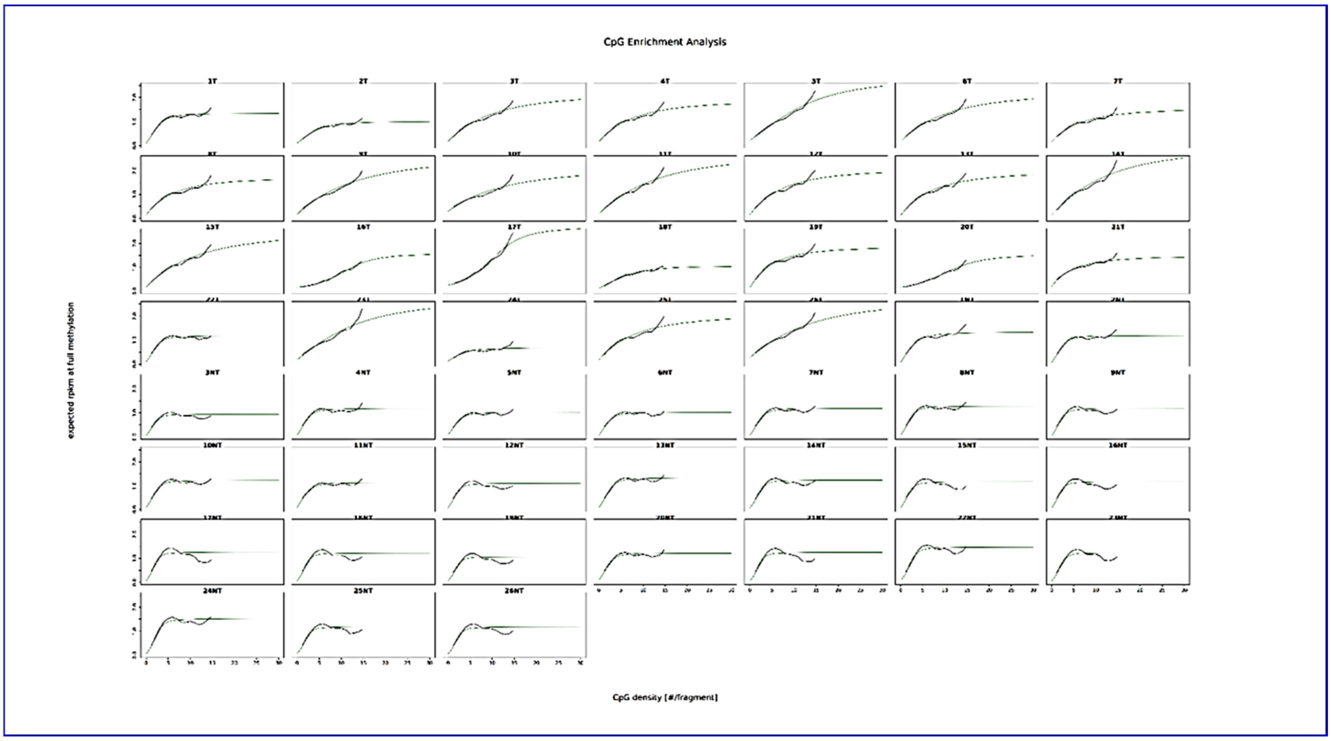

Suppl Figure 12: The summary of the CpG density in the HCC and adjacent non-tumoral tissue [HCC: Hepatocellular carcinoma].

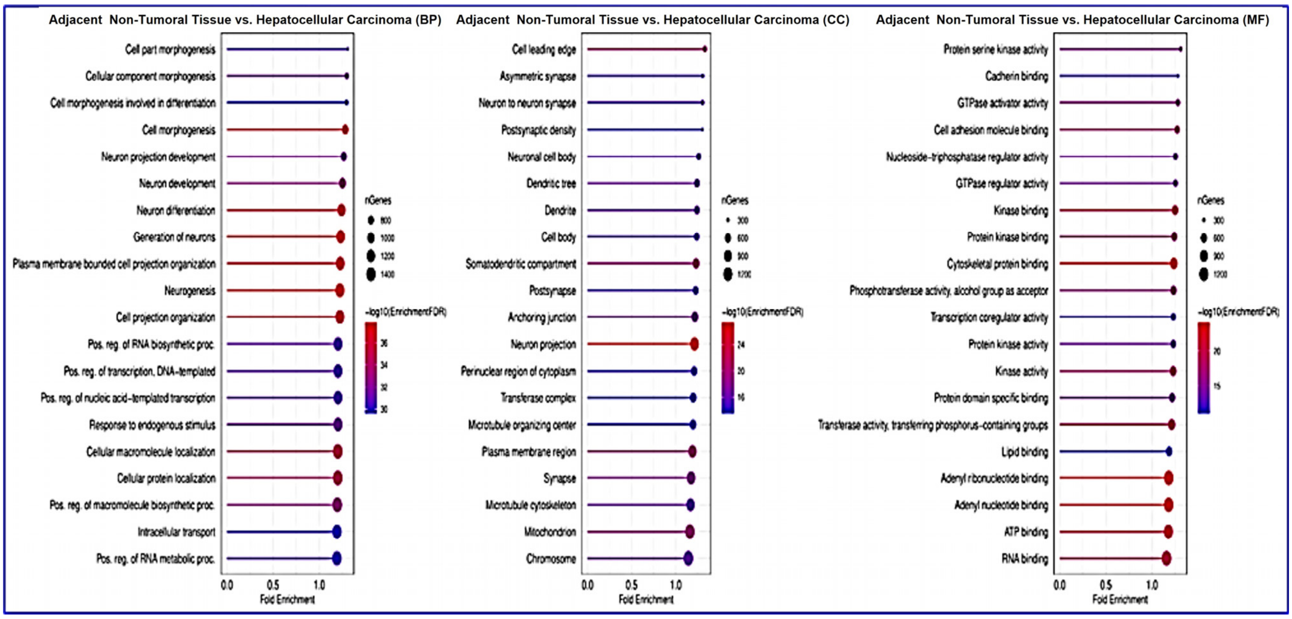

Suppl Figure 13: The summary of the GO functional annotation of the gene loci that showed differences in methylation profile determined by MEDIP-Seq analysis in the HCC tissues compared to the adjacent non-tumoral tissues. [GO: gene ontology, BP: biologic process, MF: molecular function, CC: cellular component, HCC: Hepatocellular carcinoma].

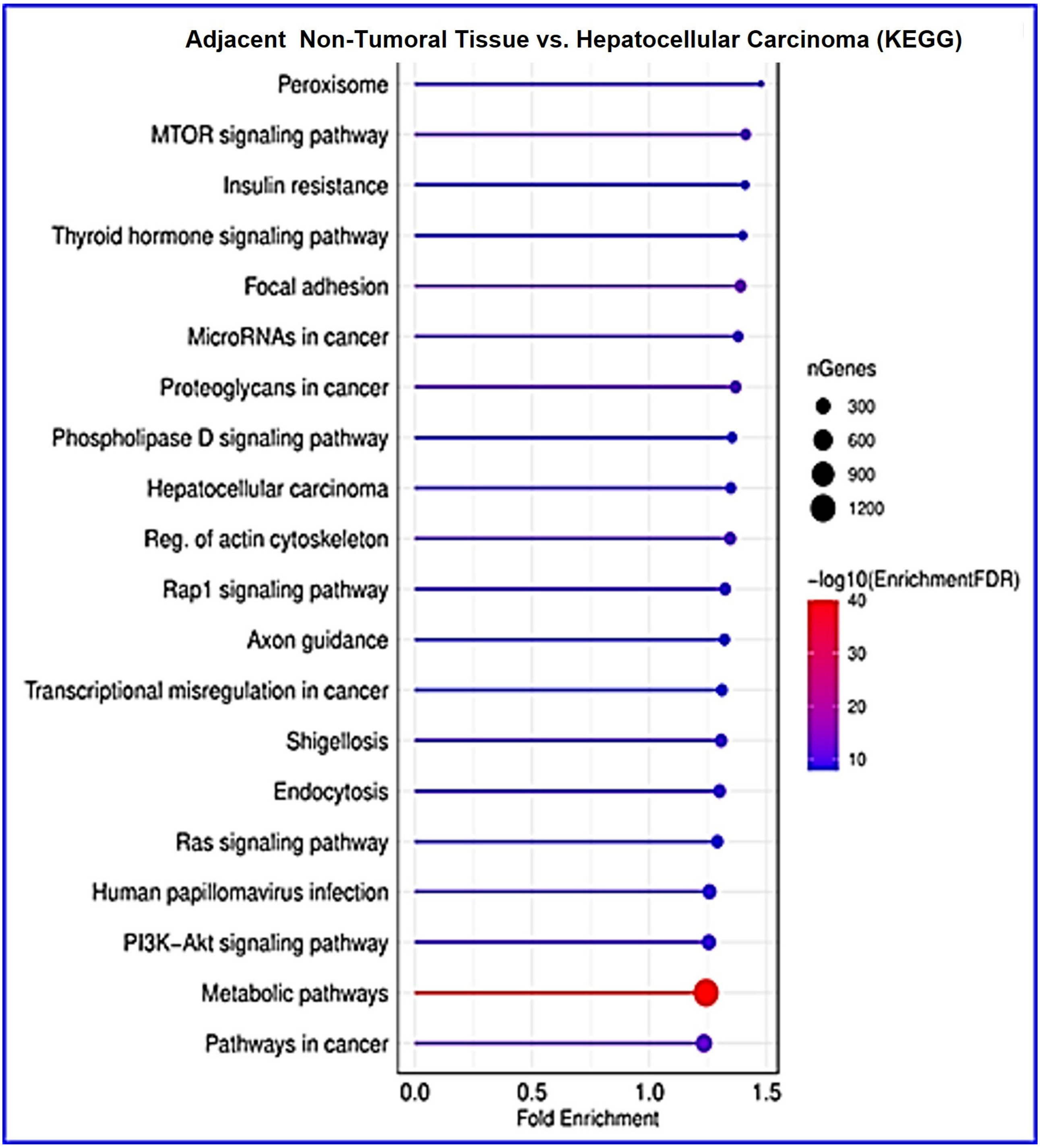

Suppl Figure 14: The summary of the KEGG metabolic pathway annotation of the gene loci that showed differences in methylation profile determined by MEDIP-Seq analyses in the HCC tissue compared to adjacent non-tumoral tissues. [KEGG: Kyoto Encyclopedia of Genes and Genomes, HCC: Hepatocellular carcinoma].
