# Supplementary material for: Role of Epigenetic Factors in Determining the Biological Behavior and Prognosis of Hepatocellular Carcinoma
Source: Diagnostics (Basel). 2024 Aug 31;14(17):1925. doi: 10.3390/diagnostics14171925 (PMC11394249; doi:10.3390/diagnostics14171925)
Supplement: Supplementary file 1 [file diagnostics-14-01925-s001.zip › Suppl Tables revised.pdf]

diagnostics

MDPI

Role of Epigenetic Factors in Determining the Biological Behavior and Prognosis of Hepatocellular Carcinoma

Suppl Table 1: The twenty-five genes that were downregulated in HCC tissues (in comparison to the non-tumoral cirrhotic tissues) in the GSE46444 data set (The genes are classified according to the adj. p value).

ID	adj.P.Val	P.Value	Log2FC	GI	GB_ACC
ILMN 2136010	5.13E-16	1.08E-20	2.2653643	NM 000670.3	ADH4
ILMN 1702383	2.93E-13	1.24E-17	1.5707109	NM 001297.1	CNGB1
ILMN 2147251	6.92E-13	4.39E-17	1.6130769	NR 001565.1	MGC10997
ILMN 1786720	1.35E-11	1.41E-15	3.0695491	NM 006017.1	PROM1
ILMN 2141650	1.35E-11	1.89E-15	1.7351626	NR 002819.1	MALAT1
ILMN 2245686	2.18E-11	3.68E-15	1.503838	NM 003918.2	GYG2
ILMN 2297096	3.42E-11	6.51E-15	1.5116106	NM 001017526.1	ARHGAP8
ILMN 1718766	4.68E-11	9.90E-15	3.0659617	NM 005949.2	MT1F
ILMN 2199389	6.40E-11	1.49E-14	2.942844	NM 004624.2	VIPR1
ILMN 1732537	6.74E-11	1.71E-14	1.1169017	NM 017802.2	HEATR2
ILMN 2346137	1.28E-10	3.53E-14	1.3014018	NM 001044387.1	ZNF557
ILMN 1714057	2.39E-10	7.06E-14	1.9520979	NM 004108.2	FCN2
ILMN 2126802	3.54E-10	1.12E-13	1.3382916	NM 015920.3	RPS27L
ILMN 2215382	9.46E-10	3.40E-13	1.046457	NM 175066.2	DDX51
ILMN 2100437	1.12E-09	4.26E-13	1.6471325	NM 000518.4	HBB
ILMN 2389851	1.79E-09	7.55E-13	1.5094376	NM 006879.2	MDM2
ILMN 2149400	6.13E-09	2.90E-12	1.3022124	NM 020675.3	SPC25
ILMN 2346562	6.13E-09	2.98E-12	1.2965792	NM 021148.2	ZNF273
ILMN 1687848	2.01E-08	1.06E-11	2.4883833	NM 000587.2	C7
ILMN 2184250	2.01E-08	1.11E-11	1.3491332	NM 004155.3	SERPINB9
ILMN 2357377	2.01E-08	1.07E-11	1.1782223	NM 003218.2	TERF1
ILMN 1683231	2.98E-08	1.76E-11	1.2859716	NM 138435.1	FAM83F
ILMN 2127416	3.52E-08	2.23E-11	1.2504523	NM 000637.2	GSR
ILMN 1751607	3.76E-08	2.54E-11	1.9227058	NM 175066.2	DDX51
ILMN 2215382	9.46E-10	3.40E-13	1.046457	NM 006732.1	FOSB

adj.P. Val: Adjusted P value; log2FC: Logarithm of fold change; GB_ACC: GenBank accession number; GI: Gene info#

Suppl Table 2: The 25 genes that were upregulated in the HCC tissues (in comparison to the non-tumoral cirrhotic tissues) in the GSE46444 data set (The genes are classified according to the adj. p-value).

ID	adj.p value	P.Value	Log2FC	GI	GB_ACC
ILMN 2131293	3.02E-07	3.19E-10	-2.794612	NM 001015050.1	ALG1L
ILMN 1674386	1.05E-05	2.49E-08	-2.3831282	NM 002653.3	PITX1
ILMN 1787266	1.10E-05	2.64E-08	-2.1452389	NM 003122.2	SPINK1
ILMN 2391458	2.39E-05	7.38E-08	-1.6111474	NM 022977.1	ACSL4
ILMN 1785252	5.95E-05	2.53E-07	-1.1433955	NM 134426.2	SLC26A6
ILMN 1757255	7.23E-05	3.28E-07	-2.0781729	NM 058197.3	CDKN2A
ILMN 1776516	9.59E-05	4.93E-07	-1.3277727	NM 002220.1	ITPKA
ILMN 1688543	1.69E-04	1.18E-06	-2.2565236	NM 001643.1	APOA2
ILMN 1717173	2.00E-04	1.52E-06	-1.0954115	NM 018098.4	ECT2
ILMN 2051373	3.01E-04	2.70E-06	-1.0149922	NM 002497.2	NEK2
ILMN 2360291	3.48E-04	3.25E-06	-1.3762846	NM 020120.2	UGCGL1
ILMN 1809590	4.23E-04	4.25E-06	-1.443501	NM 016095.1	GINS2
ILMN 1678841	4.70E-04	4.98E-06	-1.3529373	NM 006398.2	UBD
ILMN 1811238	5.94E-04	7.15E-06	-1.7252767	NM 052947.3	ALPK2
ILMN 1712803	1.16E-03	1.94E-05	-1.1197562	NM 031966.2	CCNB1
ILMN 2354140	1.44E-03	2.81E-05	-1.2402484	NM 181528.2	NAT5
ILMN 2209515	1.77E-03	3.84E-05	-2.0751683	NR 002739.1	SNORD56
ILMN 1737813	1.78E-03	3.86E-05	-1.4745061	XM 001132636.1	PRNPIP
ILMN 1756849	1.96E-03	4.48E-05	-1.5426093	NM 021052.2	HIST1H2AE
ILMN 1694268	2.17E-03	5.33E-05	-1.2911237	NM 018645.3	HES6
ILMN 1657204	2.74E-03	7.59E-05	-1.9435086	NM 005500.1	SAE1
ILMN 2322346	2.82E-03	7.89E-05	-1.032828	NM 175729.1	SSX4
ILMN 1806692	2.83E-03	7.95E-05	-1.2883191	NM 000521.2	HEXB
ILMN 1777725	2.85E-03	8.06E-05	-1.2067761	NM 144703.2	LSM14B
ILMN 2409220	3.16E-03	9.49E-05	-1.1261905	NM 012484.1	HMMR

adj.P.Val: Adjusted P value; log2FC: Logarithm of fold change; GB_ACC: GenBank accession number; GI: Gene info

Suppl Table3: The results of the enriched biologic process analyses of the 25 genes that were downregulated in HCC (in comparison to the non-tumoral cirrhotic tissues) in GSE46444 data set. (Data are classified according to the FDR).

Enrichment FDR	Number of Genes	Pathway Genes	Fold Enrichment	Pathway
4.05201737300478e-08	17	118	8.31435314310234	Long-chain fatty acid metabolic proc.
9.25316106971073e-12	24	167	8.29385280072766	Olefinic compound metabolic proc.
1.34295259215676e-07	16	117	7.89215622633344	Xenobiotic metabolic proc.
6.2258575108948e-08	17	125	7.84874936708861	Cellular response to xenobiotic stimulus
1.36589941640796e-07	16	119	7.75951494521859	Unsaturated fatty acid metabolic proc.
1.34295259215676e-07	17	135	7.26736052508204	Response to xenobiotic stimulus
2.5842886292527e-07	17	147	6.67410660466718	Detoxification
8.23416771299791e-08	19	166	6.605520817447	Cellular hormone metabolic proc.
4.04755935098164e-10	27	264	5.90230149597238	Response to toxic substance
3.0313955234349e-07	22	259	4.90212599579688	Hormone metabolic proc.
2.72954403676217e-07	35	616	3.27905638665132	Cellular metal ion homeostasis
2.5842886292527e-07	37	675	3.16343928738865	Monocarboxylic acid metabolic proc.
2.57108042818129e-07	38	704	3.11510356731876	Cellular cation homeostasis
3.40780778278899e-07	38	719	3.05011531487122	Cellular ion homeostasis
2.09372550098576e-07	50	1102	2.61848422890487	Carboxylic acid metabolic proc.
2.0886503920785e-07	51	1135	2.59319913009535	Oxoacid metabolic proc.
4.46401863280507e-07	51	1178	2.49854075777439	Cellular lipid metabolic proc.
4.08839576313674e-08	65	1555	2.41237331596728	Lipid metabolic proc.
1.36589941640796e-07	72	1911	2.17436957256125	Homeostatic proc.

FDR: False discovery rate

Suppl Table 4: The results of the enriched biologic process analyses of the 25 genes that were up regulated in HCC (in comparison to the non-tumoral cirrhotic tissues) in GSE46444 data set. (Data are classified according to the FDR).

Enrichment FDR	Number of Genes	Pathway Genes	Fold Enrichment	Pathway
0.00106176328850607	3	4	43.2835443037975	Anandamide 8,9 epoxidase activity
7.99508555318435e-05	6	19	18.2246502331779	NAD-retinol dehydrogenase activity
0.000500398393326624	5	17	16.9739389426657	Arachidonic acid epoxygenase [illegible]
0.000108692002131146	6	21	16.4889692585895	Arachidonic acid monooxygenase [illegible]
0.000486429621850252	6	28	12.3667269439421	Aromatase activity
8.52970616772582e-06	9	43	12.0791286429202	Steroid hydroxylase activity
9.31379282575115e-05	8	44	10.4929804372842	Oxidoreductase activity
0.000327128546244309	7	39	10.3584550470626	Oxygen binding
0.000114853350878558	8	47	9.82321572852141	Oxidoreductase activity
0.0011197848066443	7	50	8.07959493670886	Cholesterol binding
1.42658412496162e-06	15	121	7.15430484360289	Monooxygenase activity
0.000327128546244309	10	92	6.27297743533297	Alcohol binding
5.97804899805608e-06	16	159	5.80743571371706	Heme binding
8.52970616772582e-06	16	169	5.46380046438469	Tetrapyrrole binding
2.32770318864223e-05	16	184	5.01838194826637	Oxidoreductase activity
0.000500398393326624	13	169	4.43933787731256	Iron ion binding
0.00106176328850607	12	158	4.38314372696683	Oxidoreductase activity
1.9822809866189e-07	43	835	2.97196392026074	Oxidoreductase activity
0.000102105757369892	46	1238	2.14436514590704	Transition metal ion binding

FDR: False discovery rate

Suppl Table 5: The results of the enriched cellular component analyses of the 25 genes that were down-regulated in HCC (in comparison to the non-tumoral cirrhotic tissues) in GSE46444 data set. (Data are classified according to the FDR).

Enrichment FDR	Number of Genes	Pathway Genes	Fold Enrichment	Pathway
0.0111323775796193	3	7	24.7334538878843	Membrane attack complex
0.0299607067686291	4	26	8.87867575462512	Pore complex
0.00772304071991529	9	105	4.94669077757685	Collagen trimer
0.0253756528432589	8	105	4.3970584689572	Azurophil granule lumen
0.00578731285408867	12	170	4.07374534623976	Primary lysosome
0.00578731285408867	12	170	4.07374534623976	Azurophil granule
0.0284165688285856	9	140	3.71001808318264	Blood microparticle
0.042853519730291	9	155	3.35098407513271	Peroxisome
0.042853519730291	9	155	3.35098407513271	Microbody
0.0270927732669155	11	193	3.28925034433003	Dendritic spine
0.0282351069112928	11	196	3.23890467579437	Neuron spine
0.0284165688285856	16	368	2.50919097413319	Secretory granule lumen
0.028974042262691	16	372	2.48221042602423	Cytoplasmic vesicle lumen
0.028974042262691	16	374	2.46893657347864	Vesicle lumen
0.0184971584737132	19	446	2.45855707555202	Collagen-containing extracellular matrix
0.00578731285408867	25	600	2.40464135021097	Extracellular matrix
0.00578731285408867	25	601	2.40064028307252	External encapsulating structure
0.00578731285408867	56	1881	1.71814884353192	Integral component of plasma membrane
0.0111323775796193	3	7	24.7334538878843	Membrane attack complex

FDR: False discovery rate

Suppl Table 6: The results of the enriched cellular component analyses of the 25 genes that were up-regulated in HCC (in comparison to the non-tumoral cirrhotic tissues) in GSE46444 data set. (Data are classified according to the FDR).

Enrichment FDR	Number of Genes	Pathway Genes	Fold Enrichment	Pathway
3.28113110467977e-09	14	69	11.7095578792882	Drug metabolism
2.95481322569978e-08	12	60	11.5422784810127	Mineral absorption
1.32678402894817e-08	13	68	11.0330603127327	Retinol metabolism
4.47350117198117e-09	14	74	10.9183715360931	Metabolism of xenobiotics by cytochrome P450
1.24247171523338e-05	10	68	8.48696947133284	Chemical carcinogenesis
0.00295632814506478	6	43	8.05275242861348	Fatty acid degradation
0.00817325820084135	5	36	8.0154711673699	Tyrosine metabolism
0.0225909884358528	4	29	7.9601920558708	Linoleic acid metabolism
0.02176426644044	5	47	6.13950983032588	Pyruvate metabolism
0.00383058285715991	7	67	6.02954846023049	Glycolysis / Gluconeogenesis
0.00295632814506478	8	82	5.63037974683544	Peroxisome
0.00383058285715991	8	89	5.18754089034277	Bile secretion
0.0123016356796435	7	84	4.80928270042194	Complement and coagulation cascades
0.0228905546118618	6	73	4.74340211548465	Platinum drug resistance
0.0160805935520511	8	115	4.0147055586131	Carbon metabolism
0.0225909884358528	9	155	3.35098407513271	Biosynthesis of cofactors
0.00903605584630444	16	350	2.63823508137432	Neuroactive ligand-receptor interaction
0.0225063400029033	15	354	2.44539798326539	PI3K-Akt signaling pathway
0.00383058285715991	22	530	2.39556723190829	Pathways in cancer
4.74534771656847e-08	61	1527	2.30543217859126	Metabolic pathways

FDR: False discovery rate

Suppl Table 7: The twenty-five genes that were downregulated in HCC tissues (in comparison to the non-tumoral cirrhotic tissues) in the GSE63898 data set (The genes are classified according to the adj. p value).

ID	adj. p value	P.Value	Log2FC	GB_ACC	GI
11717912_s_at	1.14E-135	2.32E-140	4.52	CXCL14	NM 004887
11731275_at	1.07E-131	4.31E-136	3.72	CLEC4G	NM 198492
11728139_a_at	2.34E-128	1.42E-132	2.61	ADAMTS13	NM 139025
11756838_a_at	1.34E-127	1.08E-131	3.96	FCN2	NM 004108
11756059_a_at	1.53E-127	1.55E-131	4.3	CXCL14	NM 004887
11731276_x_at	5.04E-110	6.13E-114	2.98	CLEC4G	NM 198492
11736982_at	4.51E-108	6.39E-112	3.27	COLEC10	NM 006438
11725216_s_at	2.58E-104	4.18E-108	4.34	FCN3	NM 003665
11725200_a_at	1.47E-101	2.69E-105	1.54	PTH1R	NM 000316
11724619_at	9.52E-99	1.93E-102	1.88	RSPO3	NM 032784
11723068_at	4.65E-96	1.13E-99	3.51	CRHBP	NM 001882
11735149_at	3.04E-95	7.99E-99	1.68	ANGPTL6	NM 031917
11722687_a_at	4.49E-94	1.36E-97	2.09	LIFR	NM 001127671
11729824_at	4.54E-91	1.56E-94	1.69	BMPER	NM 133468
11733298_a_at	2.15E-90	7.85E-94	1.66	VIPR1	NM 004624
11717301_at	3.03E-90	1.17E-93	3.79	TACSTD2	NM 002353
11756904_a_at	1.13E-89	4.56E-93	1.72	STAB2	NM 017564
11732904_a_at	6.22E-87	2.64E-90	2.68	CLEC1B	NM 001099431
11717891_a_at	3.34E-86	1.49E-89	1.6	ECM1	NM 004425
11720818_a_at	3.77E-86	1.75E-89	3.09	CXCL12	NM 000609
11754563_a_at	6.96E-86	3.38E-89	1.44	CCBE1	NM 133459
11742268_x_at	8.48E-85	4.30E-88	2.54	CLEC4M	NM 001144904
11753257_a_at	4.54E-83	2.57E-86	2.89	CXCL12	NM 000609
11752118_x_at	1.01E-82	5.93E-86	1.42	TBXA2R	NM 001060
11731862_at	1.18E-82	7.39E-86	3.03	CDH19	NM 021153

adj.P.Val: Adjusted P value; log2FC: Logarithm of fold change; GB_ACC: GenBank accession number; GI: Gene info

Suppl Table 8: The twenty-five genes that were upregulated in HCC tissues (in comparison to the non-tumoral cirrhotic tissues) in the GSE63898 data set (The genes are classified according to the adj. p value).

ID	adj.P.Val	P.Value	Log2FC	GB_ACC	GI
11748436_x_at	4.43E-98	9.88E-102	-2.69	CAP2	NM 006366
11720970_at	2.83E-83	1.55E-86	-3.34	TOP2A	NM 001067
11742832_a_at	4.6E-76	4.75E-79	-2.5	ASPM	NM 018136
11750144_x_at	2.7E-74	3.17E-77	-1.07	CCT3	NM 001008800
11717664_s_at	3.68E-72	4.77E-75	-1.11	KLHL12	NM 021633
11717176_at	1.78E-71	2.45E-74	-1.1	SNX27	NM 030918
11758149_s_at	3.72E-71	5.27E-74	-1.9	RACGAP1	NM 001126103
11716920_at	4.24E-68	7.56E-71	-1.98	PLVAP	NM 031310
11721562_a_at	2.4E-67	4.51E-70	-1.37	FAM189B	NM 006589
11721480_s_at	7.77E-67	1.5E-69	-1.18	GOLPH3L	NM 018178
11758089_s_at	8.83E-67	1.72E-69	-2	HMMR	NM 001142556
11721042_at	2.99E-66	5.94E-69	-1.49	NSMCE2	NM 173685
11722711_a_at	3.09E-65	6.56E-68	-1.3	PPOX	NM 000309
11718943_a_at	2.88E-64	6.65E-67	-2.02	AURKA	NM 003600
11717521_x_at	7.83E-64	1.87E-66	-1.91	PTTG1	NM 004219
11757699_x_at	9.2E-64	2.22E-66	-1.16	PSMD4	NM 002810
11751086_x_at	1.41E-63	3.42E-66	-1.28	GBA	NM 000157
11718790_at	1.63E-63	4.03E-66	-1.63	HES6	NM 001142853
11733695_a_at	2.08E-63	5.19E-66	-2.52	UBE2C	NM 007019
11747203_x_at	7.95E-62	2.11E-64	-1.19	CLN3	NM 000086
11716336_a_at	6.07E-61	1.66E-63	-1.12	PRCC	NM 005973
11736167_a_at	5.18E-60	1.51E-62	-1.61	CCDC34	NM 030771
11755529_s_at	1.89E-59	5.78E-62	-1.01	ZNF322A	NM 024639
11757036_x_at	4.42E-59	1.38E-61	-1.2	SAC3D1	NM 013299
11716358_s_at	8.76E-59	2.78E-61	-1.98	PRC1	NM 003981

#

adj.P.Val: Adjusted P value; log2FC: Logarithm of fold change; GB_ACC: GenBank accession number; GI: Gene info

Suppl Table 9: The results of the enriched biologic process analyses of the 25 genes that were downregulated in HCC (in comparison to the non-tumoral cirrhotic tissues) in the GSE63898 data set. (Data are classified according to the FDR).

Enrichment FDR	Number of Genes	Pathway Genes	Fold Enrichment	Pathway
2.27328583057565e-17	42	167	5.57697057247373	Olefinic compound metabolic proc.
8.01936526384558e-16	57	331	3.81867234062562	Carboxylic acid biosynthetic proc.
1.98374277728846e-16	60	352	3.77984612663601	Steroid metabolic proc.
1.05262767697712e-17	68	414	3.64228650914491	Fatty acid metabolic proc.
9.78926469046677e-27	107	675	3.5151635682375	Monocarboxylic acid metabolic proc.
7.78496930260339e-16	79	584	2.9997135014125	Response to inorganic substance
4.95709241762695e-26	140	1102	2.81716299335485	Carboxylic acid metabolic proc.
2.34836366506075e-25	141	1135	2.75479182022318	Oxoacid metabolic proc.
2.39895532071157e-20	117	950	2.73103829612943	Inflammatory response
5.21275833071 85e-16	112	1017	2.44209527526218	Response to lipid
2.27328583057565e-17	142	1404	2.24278049375326	Response to cytokine
2.66757054326265e-19	163	1639	2.20533304211783	Cell adhesion
5.53782430294381e-18	154	1555	2.19611895854968	Lipid metabolic proc.
3.63236471683964e-19	163	1646	2.19595434752802	Biological adhesion
2.30940218370398e-21	181	1832	2.19088024399776	Response to oxygen-containing compound
7.15139405605581e-17	153	1590	2.1338301152632	Cell migration
2.27328583057565e-17	166	1769	2.08087402366304	Response to endogenous stimulus
2.27328583057565e-17	169	1817	2.06251592718146	Reg. of cell population proliferation

FDR: False discovery rate#

Suppl Table 10: The results of the enriched biologic process analyses of the 25 genes that were upregulated in HCC (in comparison to the non-tumoral cirrhotic tissues) in the GSE63898 data set. (Data are classified according to the FDR).

Enrichment FDR	Number of Genes	Pathway Genes	Fold Enrichment	Pathway
1.88052153197125e-09	11	17	14.34859235523	Arachidonic acid epoxygenase activity
6.53686128380926e-09	13	28	10.2955808782657	Aromatase activity
2.57951230111518e-11	18	43	9.28259885983169	Steroid hydroxylase activity
4.02368179929045e-10	17	44	8.56765122037496	Oxidoreductase activity
1.09027251286369e-07	15	47	7.07715870519083	Oxidoreductase activity
1.52188606817732e-13	32	121	5.86448853587163	Monooxygenase activity
2.22219354047808e-13	36	159	5.02077674179576	Heme binding
1.36337308035185e-12	36	169	4.72368936062441	Tetrapyrrole binding
1.96657598514269e-13	39	184	4.70015648790391	Oxidoreductase activity
3.2639667952219e-11	34	169	4.46126217392305	Iron ion binding
4.03012637803852e-10	34	186	4.05351240533869	Extracellular matrix structural constituent
9.24696014161281e-07	26	158	3.64906664039797	Oxidoreductase activity
3.16471764032381e-06	24	147	3.62042404510442	Oxidoreductase activity
1.83910810837617e-10	41	253	3.59359283923655	Glycosaminoglycan binding
1.41042115194913e-06	28	184	3.37447132464896	Heparin binding
1.26931089094021e-18	105	835	2.78848528623686	Oxidoreductase activity
2.13521043693730e-09	138	1722	1.77709838799333	Signaling receptor binding
1.52730071759966e-06	99	1238	1.77329130076752	Transition metal ion binding
1.71269713443196e-06	111	1446	1.70223775772156	Protein-containing complex binding

FDR: False discovery rate

Suppl Table 11: The results of the enriched cellular component analyses of the 25 genes that were downregulated in HCC (in comparison to the non-tumoral cirrhotic tissues) in the GSE63898 data set. (Data are classified according to the FDR).

Enrichment FDR	Number of Genes	Pathway Genes	Fold Enrichment	Pathway
4.56097746418143e-06	16	70	5.06859366314619	Platelet alpha granule lumen
1.05889557160392e-07	26	140	4.11823235130628	Blood microparticle
9.27543641718456e-05	17	100	3.76976653696498	Platelet alpha granule
3.51021940169203e-20	74	446	3.67927622969412	Collagen-containing extracellular matrix
3.17129764311454e-20	88	600	3.25234760051881	Extracellular matrix
3.17129764311454e-20	88	601	3.24693604045139	External encapsulating structure
4.56097746418143e-06	35	283	2.74250319671117	Basal part of cell
6.24340859099691e-05	29	240	2.67949092088197	Basolateral plasma membrane
2.23308458935479e-05	32	265	2.6777475956244	Basal plasma membrane
3.74396456856492e-06	42	368	2.53085349348672	Secretory granule lumen
4.45697421913458e-06	42	372	2.50364001506213	Cytoplasmic vesicle lumen
4.56097746418143e-06	42	374	2.49025156578372	Vesicle lumen
3.95971152230525e-05	36	328	2.43385214007782	Endoplasmic reticulum lumen
0.000108213454833219	37	360	2.27910722006053	Membrane raft
0.000108213454833219	37	360	2.27910722006053	Membrane microdomain
3.21432864232179e-05	42	409	2.27714935355284	External side of plasma membrane
7.98966016125633e-11	95	960	2.19441066796368	Cell surface
6.41433400553834e-06	62	677	2.03080654524137	Side of membrane
2.43598117902435e-06	104	1323	1.74316713282806	Plasma membrane region

FDR: False discovery rate

Suppl Table 12: The results of the enriched cellular component analyses of the 25 genes that were upregulated in HCC (in comparison to the non-tumoral cirrhotic tissues) in the GSE63898 data set. (Data are classified according to the FDR).

Enrichment FDR	Number of Genes	Pathway Genes	Fold Enrichment	Pathway
1.7380515489285e-11	22	68	7.17429617761502	Retinol metabolism
5.41527970637281e-07	13	43	6.70409917654511	Fatty acid degradation
6.47102366642794e-10	20	68	6.52208743419547	Chemical carcinogenesis
6.47102366642794e-10	20	69	6.42756442790278	Drug metabolism
3.93434491730006e-07	14	49	6.33574207893274	Intestinal immune network for IgA production
5.50884583270273e-10	21	74	6.29293301083184	Metabolism of xenobiotics by cytochrome P450
6.47102366642794e-10	22	84	5.80776357235501	Complement and coagulation cascades
1.75471992846654e-10	24	92	5.7848079851125	Rheumatoid arthritis
7.75271168814308e-07	15	60	5.54377431906615	Mineral absorption
3.93434491730006e-07	16	65	5.45848548338821	Inflammatory bowel disease
8.9164799716463e-07	15	61	5.45289277285195	Arachidonic acid metabolism
8.04090594697217e-10	23	94	5.42582167397963	Staphylococcus aureus infection
1.0512991075765e-07	18	75	5.3220233463035	PPAR signaling pathway
1.22572624189075e-07	18	76	5.25199672332582	Leishmaniasis
6.58848254272e-08	20	89	4.98316792725047	Bile secretion
1.01731915327503e-07	26	151	3.81822867008529	Phagosome
3.93434491730006e-07	24	141	3.77448464276844	Alcoholic liver disease
1.01731915327503e-07	43	354	2.69358526237112	PI3K-Akt signaling pathway
4.30787936263117e-08	57	530	2.38486895235298	Pathways in cancer

FDR: False discovery rate

diagnostics

MDPI

Suppl Table 13: The top 20 regions that were hypermethylated in the HCC tissues in comparison to the healthy livers.

Chr	Start	End	Length	nCG	meanMethy1	Mean Methy2	diff. Methy	Gene
Chr19	18868555	18869451	897	235	0.235186431	0.658857821	-0.42367139	GDF1.CERS1
Chr12	95548305	95548761	457	123	0.072308236	0.431109339	-0.358801103	USP44
Chr11	65585129	65585630	502	147	0.440940212	0.810706761	-0.369766549	EHBP1L1.SIPA1
Chr 6	149450761	149451029	269	76	0.08041282	0.564716028	-0.484303208	ZC3H12D
Chr 9	97854276	97854644	369	113	0.078294011	0.365391189	-0.287097178	FOXE1
Chr 2	232928064	232928356	293	98	0.143035456	0.485520992	-0.342485535	NGEF
Chr 8	144580889	144581255	367	85	0.136565013	0.488468021	-0.351903007	ARHGAP39
Chr20	51802166	51802543	378	77	0.156406622	0.543060201	-0.386653579	SALL4
Chr 2	111119083	111119594	512	102	0.140017746	0.400564632	-0.260546886	BCL2L11
Chr 1	228212371	228212665	295	89	0.239497106	0.65430122	-0.414804114	OBSCN.OBSCN-AS1
Chr11	47590085	47590397	313	93	0.224428863	0.585826515	-0.361397652	C1QTNF4
Chr10	132785114	132785445	332	74	0.13008709	0.517303457	-0.387216368	NKX6-2
Chr 2	27442370	27442622	253	60	0.202423741	0.715603692	-0.513179951	KRTCAP3
Chr13	109140491	109140939	449	98	0.183077964	0.505396277	-0.322318313	MYO16
Chr22	46262914	46263179	266	90	0.200710114	0.517110868	-0.316400754	PKDREJ
Chr14	69571638	69572053	416	94	0.09199152	0.379308554	-0.287317034	CCDC177
Chr18	14999182	14999544	363	91	0.280694541	0.587093175	-0.306398634	-
Chr X	20116894	20117130	237	85	0.216460338	0.582363595	-0.365903257	MAP7D2
Chr22	46536460	46536763	304	98	0.12393005	0.385246352	-0.261316302	CELSR1
Chr16	54931155	54931474	320	93	0.066441253	0.282466587	-0.216025334	IRX5

Chr: chromosome; nCG: The Network of Cancer Genes, Mean Methy: Mean methylation, diff. Methy: Differential methylation

Suppl Table 14: The top 20 hypomethylated gene loci in HCC tissues in comparison to the healthy livers.

Chr	Start	End	Length	nCG	Mean Methy1	Mean Methy2	diff. Methy	Gene
Chr7	54668058	54668219	162	23	0.792011913	0.208281875	0.583730039	NCR
Chr8	56653607	56653810	204	26	0.70370147	0.258297167	0.445404303	NCR
Chr4	164188238	164188326	89	21	0.77572998	0.237901844	0.537828136	ENSG00000250746
Chr9	134991017	134991130	114	20	0.753474248	0.224125421	0.529348828	NCR
Chr4	134952933	134953049	117	26	0.777062983	0.317149665	0.459913318	NCR
Chr7	118670718	118670921	204	23	0.727011693	0.299386285	0.427625408	NCR
Chr4	35368751	35368869	119	20	0.724687084	0.228252488	0.496434596	NCR
ChrY	11306809	11306901	93	18	0.811512519	0.229931177	0.581581342	NCR
Chr5	17390117	17390187	71	16	0.680504914	0.133607872	0.546897042	NCR
Chr19	39403942	39404055	114	20	0.435369296	0.043617478	0.391751818	NCR
Chr 7	19266171	19266240	70	14	0.638600143	0.095398566	0.543201577	NCR
Chr 7	72097927	72098010	84	12	0.613731923	0.143528528	0.470203395	NCR
Chr 3	84918685	84918795	111	19	0.735090196	0.295136692	0.439953504	NCR
Chr 8	67946096	67946163	68	12	0.796230086	0.238049921	0.558180165	NCR
Chr 9	20097835	20097903	69	14	0.726523435	0.186747991	0.539775444	NCR
Chr 9	80328186	80328388	203	18	0.635562924	0.219394936	0.416167988	NCR
Chr 8	141760558	141760670	113	16	0.566602633	0.139929237	0.426673396	NCR
Chr 8	91409226	91409367	142	17	0.676085431	0.137734109	0.538351321	NCR
Chr19	35371392	35372097	706	25	0.719273626	0.338686677	0.380586949	GPR42
Chr11	1194517	1194759	243	23	0.648746685	0.29458712	0.354159565	MUC5AC

Chr: chromosome; nCG: The Network of Cancer Genes, Mean Methy: Mean methylation, diff. Methy: Differential methylation, NCR: non-coding region.

diagnostics

Suppl Table 15: The summary of the biologic processes involving the genes that had different methylation profile in HCC tissues in comparison to the healthy liver tissues.

Gene number	BP Gen number	Biologic process (BP)	Fold change	FDR
35	230	Skeletal system morphogenesis	3.617	3.44253E-08
38	274	Sensory organ morphogenesis	3.297	4.98902E-08
42	307	Embryonic organ morphogenesis	3.252	1.61987E-08
37	290	Axon guidance	3.033	7.31354E-07
37	291	Neuron projection guidance	3.022	7.62446E-07
56	460	Embryonic organ development	2.894	2.14456E-09
74	622	Embryonic morphogenesis	2.828	4.28629E-12
57	527	Skeletal system development	2.571	3.80485E-08
65	641	Chordate embryonic development	2.410	3.44253E-08
66	661	Embryo development ending in birth or egg hatching	2.373	3.80485E-08
58	589	Sensory organ development	2.341	7.14944E-07
104	1102	Embryo development	2.243	2.3727E-11
100	1133	Animal organ morphogenesis	2.098	2.5053E-09
75	856	Head development	2.083	6.45516E-07
71	811	Brain development	2.081	1.48731E-06
101	1183	Anatomical structure formation involved in morphogenesis	2.029	9.50111E-09
94	1105	Central nervous system development	2.022	3.44253E-08
124	1624	Generation of neurons	1.815	3.44253E-08
134	1757	Neurogenesis	1.813	9.50111E-09
112	1473	Neuron differentiation	1.807	2.93483E-07

HCC: Hepatocellular carcinoma; FDR: False discovery rate

diagnostics MDPI

Suppl Table 16: The summary of molecular functions of the genes that have different methylation profiles in HCC tissues in comparison to the healthy liver tissues#

Gene number	MF Gene number	Molecular function (MF)	Fold	FDR
15	92	Voltage-gated potassium channel activity	3.876	0.000470357
18	128	Potassium channel activity	3.343	0.000470357
28	211	Voltage-gated ion channel activity	3.154	8.52929E-06
21	166	Potassium ion transmembrane transporter activity	3.007	0.000470357
22	186	Extracellular matrix structural constituent	2.812	0.000722617
37	365	Gated channel activity	2.410	7.40678E-05
41	459	Ion channel activity	2.123	0.00042347
111	1272	Cis-regulatory region sequence-specific DNA binding	2.074	3.93987E-11
108	1253	RNA polymerase II cis-regulatory region sequence-specific DNA binding	2.049	1.59184E-10
43	509	Channel activity	2.008	0.000722617
131	1596	Transcription cis-regulatory region binding	1.951	2.68047E-11
131	1598	Transcription regulatory region nucleic acid binding	1.949	2.68047E-11
118	1446	DNA-binding transcription factor activity. RNA polymerase II-specific	1.940	3.25729E-10
144	1767	Sequence-specific DNA binding	1.937	8.13517E-12
135	1660	Sequence-specific double-stranded DNA binding	1.933	2.68047E-11
121	1488	RNA polymerase II transcription regulatory region sequence-specific	1.933	2.35252E-10
123	1515	DNA-binding transcription factor activity	1.930	1.90855E-10
60	770	Calcium ion binding	1.852	0.000302319
136	1775	Double-stranded DNA binding	1.821	5.04506E-10

HCC: Hepatocellular carcinoma; FDR: False discovery rate; MF: Molecular function

diagnostics MDPI

Suppl Table 17: The summary of the cellular components of the genes that had a different methylation profile in HCC tissues in comparison to the healthy liver tissues.

Gene number	CC Gene number	Cellular component	Fold change	FDR
9	63	Anchored component of plasma membrane	3.396	0.046906355
10	72	Main axon	3.301	0.037635138
13	94	Voltage-gated potassium channel complex	3.287	0.013040281
13	104	Potassium channel complex	2.971	0.026466861
22	238	Cation channel complex	2.197	0.026466861
29	324	Ion channel complex	2.128	0.011246
25	297	Postsynaptic membrane	2.001	0.037635138
33	401	Synaptic membrane	1.956	0.015576207
106	1316	Chromatin	1.915	5.94107E-08
46	600	Extracellular matrix	1.822	0.008050889
46	601	External encapsulating structure	1.819	0.008050889
34	445	Cell leading edge	1.816	0.032406579
103	1443	Synapse	1.697	2.55204E-05
48	682	Post-synapse	1.673	0.025763777
48	712	Plasma membrane protein complex	1.603	0.040021777
128	1918	Chromosome	1.586	2.55204E-05
58	907	Anchoring junction	1.520	0.042258906
125	1965	Intrinsic component of plasma membrane	1.512	0.000343825
116	1881	Integral component of plasma membrane	1.466	0.002777085

HCC: Hepatocellular carcinoma; FDR: False discovery rate; CC: Cellular component

#

Suppl Table 18: The summary of the enriched metabolic pathway analyses of the genes that showed different methylation profiles in HCC tissues in comparison to the healthy liver tissues [KEGG: Kyoto Encyclopedia of Genes and Genomes].

Gene number	KEGG Gene number	Metabolic pathway	Fold change	FDR
10	63	Basal cell carcinoma	3.773	0.025788425
12	88	ECM-receptor interaction	3.241	0.025788425
16	143	Signaling pathways regulating pluripotency of stem cells	2.660	0.025788425
17	155	Cushing syndrome	2.607	0.025788425
39	530	Pathways in cancer	1.749	0.032382387
10	63	Basal cell carcinoma	3.773	0.025788425

HCC: Hepatocellular carcinoma; FDR: False discovery rate; KEGG: Kyoto Encyclopedia of Genes and Genomes

diagnostics MDPI

Suppl Table 19: The top 20 gene loci that were hypermethylated in HCC tissues in comparison to the cirrhotic liver tissues.

Chr	Start	End	Length	nCG	meanMethy1	meanMethy2	diff.Methy	Gene
Chr2	232927897	232928396	500	145	0.063467253	0.440913095	-0.377445842	NGEF
Chr12	95548305	95548761	457	123	0.043876472	0.431241542	-0.38736507	USP44
Chr 6	43076260	43076765	506	109	0.033236281	0.377256203	-0.344019922	PTK7
Chr 7	128031530	128032060	531	106	0.121119756	0.495425774	-0.374306018	LRRC4
Chr 8	144580896	144581255	360	79	0.106389766	0.49165857	-0.385268804	ARHGAP39
Chr 3	125141679	125142086	408	92	0.06698527	0.422539198	-0.355553928	NCR
Chr 9	131276713	131276935	223	82	0.05783991	0.438544814	-0.380704904	NCR
Chr 1	151839734	151840074	341	64	0.055725076	0.468484749	-0.412759673	NCR
Chr 7	151115171	151115380	210	73	0.089418907	0.45829101	-0.368872103	AGAP3
Chr21	31558961	31559197	237	72	0.065122375	0.488994271	-0.423871896	TIAM1
Chr 7	128031127	128031456	330	74	0.148062111	0.556800139	-0.408738028	NCR
Chr17	44325315	44325712	398	75	0.07324964	0.411974315	-0.338724675	NCR
Chr13	109140599	109140996	398	85	0.168588199	0.521719623	-0.353131424	MYO16
Chr 7	151115563	151115820	258	77	0.260565772	0.624263944	-0.363698172	AGAP3
Chr17	19579914	19580306	393	71	0.046267031	0.38785507	-0.341588039	SLC47A1P1
Chr20	51802110	51802438	329	69	0.187718991	0.524781834	-0.337062843	SALL4
Chr20	1803684	1803906	223	62	0.056377936	0.512393429	-0.456015493	NCR
Chr 8	144266539	144266767	229	62	0.129486832	0.542830132	-0.413343301	SCX
Chr 3	179451236	179451384	149	56	0.061239652	0.450465669	-0.389226017	GNB4
Chr12	122183103	122183286	184	54	0.070963497	0.483481567	-0.41251807	LRRC43

HCC: Hepatocellular carcinoma; Chr: chromosome; nCG: The Network of Cancer Genes; Mean Methy: Mean methylation, diff. Methy: Differential methylation

Suppl Table 20: The top 20 gene loci that were hypomethylated in HCC tissues in comparison to the cirrhotic liver tissues.

Chr	Start	End	Length	nCG	meanMethy1	meanMethy2	diff.Methy	Gene
Chr 5	20305514	20305688	175	39	0.727918033	0.320174941	0.407743092	CDH18-AS1
Chr 3	84918681	84918867	187	25	0.753673619	0.275345976	0.478327642	NCR
Chr 2	2186148	2186257	110	20	0.753486833	0.278419228	0.475067605	NCR
Chr 22	48750918	48751051	134	31	0.771046479	0.405988259	0.36505822	TAFA5
Chr 8	56653617	56653809	193	23	0.73906471	0.266799066	0.472265644	NCR
Chr 9	79193660	79193747	88	28	0.793387747	0.432624033	0.360763714	NCR
Chr14_KI270724	2179	2332	154	23	0.708832338	0.246975489	0.461856848	NCR
Chr 4	164188238	164188327	90	22	0.779664509	0.235855584	0.543808925	ENSG00000250746
Chr 6	121682813	121682904	92	24	0.871247676	0.506556164	0.364691513	-
Chr 12	4370512	4370575	64	19	0.787795859	0.293302008	0.494493851	FGF23
Chr 20	24543273	24543453	181	24	0.731130853	0.329728969	0.401401885	SYNDIG1
Chr 7	158570677	158570760	84	21	0.700400634	0.301015809	0.399384825	NCR
Chr 4	54890886	54890961	76	18	0.834119604	0.399976716	0.434142888	NCR
Chr 12	132260938	132261048	111	26	0.729009305	0.373334188	0.355675117	GALNT9
Chr 17	83134645	83134718	74	20	0.839168489	0.420145925	0.419022564	ENSG00000279143
Chr 11	130473415	130473568	154	23	0.777456089	0.452285251	0.325170838	ADAMTS15
Chr 8	141760558	141760622	65	14	0.626166697	0.131249072	0.494917625	NCR
Chr 18	61071276	61071336	61	15	0.839575317	0.372255641	0.467319676	NCR
Chr 4	28811954	28812018	65	16	0.745237908	0.256611788	0.488626119	NCR
Chr 4	138854078	138854144	6 7	13	0.777092677	0.300290781	0.476801895	NCR

HCC: Hepatocellular carcinoma; Chr: chromosome; nCG: The Network of Cancer Genes; Mean Methy: Mean methylation, diff. Methy: Differential methylation

Suppl Table 21: The summary of the biologic process analyses of the gene loci that showed different methylation profiles in HCC tissues compared to the cirrhotic liver tissues.

Gene number	BP Gene number	Biologic process	Fold change	FDR
26	225	Reg. of synapse organization	3.599	6.34298E-06
27	236	Reg. of synapse structure or activity	3.563	4.81566E-06
31	307	Embryonic organ morphogenesis	3.145	6.34298E-06
45	451	Synapse organization	3.107	1.2496E-08
44	460	Embryonic organ development	2.979	6.25194E-08
46	490	Axonogenesis	2.924	4.80488E-08
62	686	Neuron projection morphogenesis	2.815	4.43868E-10
48	533	Axon development	2.805	6.57326E-08
63	700	Plasma membrane bounded cell projection morphogenesis	2.803	4.43868E-10
63	704	Cell projection morphogenesis	2.787	4.43868E-10
55	620	Cell morphogenesis involved in neuron differentiation	2.763	9.50396E-09
63	721	Cell part morphogenesis	2.721	7.66194E-10
68	815	Cellular component morphogenesis	2.598	7.66194E-10
63	757	Cell junction organization	2.592	5.30338E-09
63	775	Cell morphogenesis involved in differentiation	2.532	1.0837E-08
77	1085	Cell morphogenesis	2.210	3.34936E-08
73	1045	Neuron projection development	2.175	1.59135E-07
71	1102	Embryo development	2.006	6.34298E-06
94	1624	Generation of neurons	1.803	6.34298E-06
94	1649	Plasma membrane bounded cell projection organization	1.775	1.07248E-05

HCC: Hepatocellular carcinoma; FDR: False discovery rate, BP: Biologic process

Suppl Table 22: The summary of the molecular function analyses of the gene loci that showed different methylation profile in HCC tissues in comparison to the cirrhotic liver tissues..

Gene number	MF Gene number	Molecular function	Fold change	FDR
17	148	Voltage-gated cation channel activity	3.577	0.000346776
21	211	Voltage-gated ion channel activity	3.099	0.000334635
31	350	Cation channel activity	2.758	5.03294E-05
29	365	Gated channel activity	2.474	0.000429512
34	454	Metal ion transmembrane transporter activity	2.332	0.000334635
34	459	Ion channel activity	2.307	0.000354662
37	509	Channel activity	2.264	0.000297826
37	509	Passive transmembrane transporter activity	2.264	0.000297826
77	1272	Cis-regulatory region sequence-specific DNA binding	1.885	1.32545E-05
75	1253	RNA polymerase II cis-regulatory region sequence-specific DNA binding	1.864	2.36264E-05
85	1488	RNA polymerase II transcription regulatory region sequence-specific DNA	1.779	2.36264E-05
91	1596	Transcription cis-regulatory region binding	1.776	1.32545E-05
91	1598	Transcription regulatory region nucleic acid binding	1.773	1.32545E-05
94	1660	Sequence-specific double-stranded DNA binding	1.763	1.32545E-05
100	1767	Sequence-specific DNA binding	1.762	1.32545E-05
59	1050	Cytoskeletal protein binding	1.750	0.001175395
81	1446	DNA-binding transcription factor activity. RNA polymerase II-specific	1.744	7.3137E-05
83	1515	DNA-binding transcription factor activity	1.706	0.000116846
95	1775	Double-stranded DNA binding	1.667	6.33012E-05
17	148	Voltage-gated cation channel activity	3.577	0.000346776

HCC: Hepatocellular carcinoma; FDR: False discovery rate; MF: Molecular function #

Suppl Table 23: The summary of the cellular component analyses of the gene loci that showed different methylation profile in HCC tissues in comparison to the cirrhotic liver tissues..

Gene number	CC Gene number	Cellular component	Fold change	FDR
4	6	Dentate gyrus mossy fiber	20.761	0.001411971
8	47	Voltage-gated calcium channel complex	5.301	0.004244293
15	143	Sarcolemma	3.267	0.002953484
55	691	Axon	2.479	2.2162E-07
23	297	Postsynaptic membrane	2.412	0.00414515
30	445	Cell leading edge	2.099	0.004244293
35	535	Neuronal cell body	2.037	0.002953484
39	614	Cell body	1.978	0.002657039
91	1444	Neuron projection	1.963	2.2162E-07
37	600	Extracellular matrix	1.920	0.004244293
42	682	Postsynapse	1.918	0.002657039
37	601	External encapsulating structure	1.917	0.004244293
56	913	Somatodendritic compartment	1.910	0.000399709
78	1323	Plasma membrane region	1.836	2.86266E-05
83	1443	Synapse	1.791	2.86266E-05
62	1110	Supramolecular polymer	1.739	0.001411971
73	1316	Chromatin	1.727	0.000414843
61	1102	Supramolecular fiber	1.724	0.001918517
95	1965	Intrinsic component of plasma membrane	1.506	0.002446255
90	1881	Integral component of plasma membrane	1.490	0.003861555

HCC: Hepatocellular carcinoma; FDR: False discovery rate; CC: Cellular Component

Suppl Table 24: The top 25 protein-coding gene loci that were hypermethylated in HCC tissues in comparison to the adjacent non-tumoral liver tissue.

Chromosome number	Window start	Window end	CpG density	Gene	TvN-log2FC	TvN p-value	TvN adj.P.Val
Chr 11	68010001	68010500	4.103108	ALDH3B1	2.573615538	1.33919E-09	2.2043E-07
Chr 11	68313501	68314000	3.951747234	LRP5	2.548263132	3.49252E-09	4.82612E-07
Chr 9	134375001	134375500	2.273332617	RXRA	2.431905946	2.79862E-21	3.69828E-17
Chr 2	240872001	240872500	4.28655468	AGXT	2.399341327	4.0509E-19	2.65266E-15
Chr X	145821001	145821500	2.201302751	SLITRK2	2.364927421	2.76221E-32	8.52987E-27
Chr 1	204363501	204364000	4.418117434	PLEKHA6	2.323727156	7.0765E-14	6.52113E-11
Chr 5	134925001	134925500	3.381178616	PCBD2	2.319114753	8.10166E-24	2.59754E-19
Chr 11	68033501	68034000	4.221942058	NDUFS8	2.314259026	1.34959E-07	9.27821E-06
Chr 1	176904001	176904500	3.283921366	ASTN1	2.155802052	1.05093E-17	4.10802E-14
Chr 1	204143501	204144000	2.068122908	ETNK2	2.153498413	3.63057E-18	1.68526E-14
Chr 7	157916501	157917000	5.314443405	PTPRN2	2.14190611	9.27495E-17	2.53466E-13
Chr 11	68716501	68717000	3.605925123	TESMIN	2.130736717	3.0391E-07	1.77678E-05
Chr 1	213995001	213995500	1.182082448	PROX1	2.123998618	6.18907E-18	2.64481E-14
Chr 11	68081501	68082000	2.936561142	CHKA	2.1164494	9.87008E-06	0.000277105
Chr 11	73140501	73141000	4.832284435	FCHSD2	2.11099418	5.6107E-08	4.58128E-06
Chr 9	125549501	125550000	2.108468335	MAPKAP1	2.109949688	6.2462E-20	5.45048E-16
Chr 14	105476001	105476500	4.435575077	CRIP2	2.108203501	7.23857E-15	9.72095E-12
Chr 11	68209001	68209500	2.050620746	KMT5B	2.096560711	2.69417E-07	1.61348E-05
Chr 1	161237501	161238000	3.211849038	NR1I3	2.085214459	1.95228E-17	6.92122E-14
Chr 21	31968501	31969000	1.511949717	HUNK	2.07778528	2.44048E-22	4.53135E-18
Chr 7	139776001	139776500	2.741003683	HIPK2	2.074895034	1.766E-12	9.47567E-10
Chr 1	225817001	225817500	2.429290459	EPHX1	2.067564788	1.40215E-15	2.44063E-12
Chr 11	2379001	2379500	2.235250164	CD81	2.05622804	9.56585E-15	1.22492E-11
Chr 1	220881001	220881500	6.619039873	MTARC1	2.055136637	1.935E-13	1.50474E-10
Chr 1	16145501	16146000	3.103902137	EPHA2	2.042039815	6.34419E-18	2.70084E-14

HCC: Hepatocellular carcinoma; Chr: chromosome; adj.P.Val: Adjusted P value; log2FC: Logarithm of fold change

Suppl Table 25: The top 25 protein-coding gene loci that were hypomethylated in HCC tissues compared to the adjacent non-tumoral liver tissue.

Chromosome number	Window start	Window end	CpG density	Gene	TvN-log2FC	TvN p-value	TvN adj.P.Val
Chr 4	97868501	97869000	0.907064718	STPG2	-2.429825333	1.67989E-34	9.85642E-29
Chr 1	95146001	95146500	0.247935351	TLCD4	-2.373610585	6.54675E-34	2.92013E-28
Chr 17	4916001	4916500	0.775683177	CHRNE	-2.238549308	1.20728E-25	7.08352E-21
Chr 8	26801501	26802000	1.164102419	ADRA1A	-2.186382299	1.0463E-25	6.26425E-21
Chr 8	18395501	18396000	0.482041218	NAT2	-2.167291012	2.16557E-24	8.47073E-20
Chr 5	36275001	36275500	0.544113417	RANBP3L	-2.153139582	3.60237E-21	4.62499E-17
Chr 10	92132001	92132500	0.927199526	CPEB3	-2.152741567	4.1576E-23	1.02496E-18
Chr 6	151926001	151926500	0.233334795	ESR1	-2.14981436	3.01071E-15	4.6474E-12
Chr 12	32867501	32868000	0.508050206	PKP2	-2.132833858	8.60117E-21	9.81824E-17
Chr 10	95053001	95053500	0.984260838	CYP2C8	-2.129297197	7.64939E-28	8.46818E-23
Chr 16	1791501	1792000	11.10055556	SPSB3	-2.11861501	4.70562E-50	2.76093E-43
Chr 4	22718001	22718500	0.769204949	GBA3	-2.113837097	2.13175E-20	2.20187E-16
Chr 12	21593001	21593500	0.402353251	GYS2	-2.108638012	2.42245E-19	1.72911E-15
Chr 4	128130001	128130500	0.747440009	LARP1B	-2.099437852	1.11088E-23	3.37715E-19
Chr 8	18672501	18673000	0.799140255	PSD3	-2.085673646	4.11359E-25	2.11717E-20
Chr 4	184784001	184784500	0.77118852	ACSL1	-2.078916279	2.1856E-22	4.08505E-18
Chr 3	51059001	51059500	0.67822644	DOCK3	-2.075652039	2.17982E-21	3.03793E-17
Chr 15	71666501	71667000	0.495584212	THSD4	-2.071990892	1.00077E-24	4.51681E-20
Chr 10	5174501	5175000	0.487761683	AKR1C8P	-2.071479179	6.23352E-24	2.1264E-19
Chr 9	13113001	13113500	1.060132359	MPDZ	-2.066597013	1.05737E-22	2.21568E-18
Chr 13	29773501	29774000	0.493225189	UBL3	-2.06659615	1.2207E-23	3.61729E-19
Chr 17	3468001	3468500	1.114134459	SPATA22	-2.064531443	7.10369E-24	2.35374E-19
Chr 8	26817001	26817500	0.758446303	FUT10	-2.062545899	5.16231E-21	6.284E-17
Chr 8	33407001	33407500	0.745039673	AADAT	-2.058459098	4.34299E-20	3.96911E-16
Chr 4	170067001	170067500	0.8271594	SCP2	-2.058241178	8.87467E-23	1.89347E-18

HCC: Hepatocellular carcinoma; Chr: chromosome; adj.P.Val: Adjusted P value; log2FC: Logarithm of fold change.
